# Nitric oxide for the prevention and treatment of viral, bacterial, protozoal and fungal infections

**DOI:** 10.12688/f1000research.51270.1

**Published:** 2021-07-05

**Authors:** Philip M. Bath, Christopher M. Coleman, Adam L. Gordon, Wei Shen Lim, Andrew J. Webb

**Affiliations:** 1 Stroke Trials Unit, Division of Clinical Neuroscience, University of Nottingham, Nottingham, Notts, NG7 2UH, UK; 2 Stroke, Nottingham University Hospitals NHS Trust, Nottingham, Notts, NG7 2UH, UK; 3 Division of Infection, Immunity and Microbes, School of Life Sciences, University of Nottingham, Nottingham, Notts, NG7 2UH, UK; 4 Unit of Injury, Inflammation and Recovery Sciences, University of Nottingham, Derby, Derbyshire, DE22 3NE, UK; 5 NIHR Applied Research Collaboration-East Midlands (ARC-EM), Nottingham, Notts, UK; 6 Respiratory Medicine, Nottingham University Hospitals NHS Trust, Nottingham, NG5 1PB, UK; 7 Clinical Pharmacology, School of Cardiovascular Medicine & Sciences, Kings College London British Heart Foundation Centre of Research Excellence, St Thomas' Hospital, London, SE1 7EH, UK

**Keywords:** Bacteria, COVID-19, fungus, nitric oxide, nitrate, nitrite, protozoa, virus

## Abstract

Although the antimicrobial potential of nitric oxide (NO) is widely published, it is little used clinically. NO is a key signalling molecule modulating vascular, neuronal, inflammatory and immune responses. Endogenous antimicrobial activity is largely mediated by high local NO concentrations produced by cellular inducible nitric oxide synthase, and by derivative reactive nitrogen oxide species including peroxynitrite and S-nitrosothiols. NO may be taken as dietary substrate (inorganic nitrate, L-arginine), and therapeutically as gaseous NO, and transdermal, sublingual, oral, intranasal and intravenous nitrite or nitrate. Numerous preclinical studies have demonstrated that NO has generic static and cidal activities against viruses (including β-coronaviruses such as SARS-CoV-2), bacteria, protozoa and fungi/yeasts *in vitro*. Therapeutic effects have been seen in animal models *in vivo*, and phase II trials have demonstrated that NO donors can reduce microbial infection. Nevertheless, excess NO, as occurs in septic shock, is associated with increased morbidity and mortality. In view of the dose-dependent positive and negative effects of NO, safety and efficacy trials of NO and its donors are needed for assessing their role in the prevention and treatment of infections. Trials should test dietary inorganic nitrate for pre- or post-exposure prophylaxis and gaseous NO or oral, topical or intravenous nitrite and nitrate for treatment of mild-to-severe infections, including due to SARS-CoV-2 (COVID-19). This review summarises the evidence base from *in vitro, in vivo* and early phase clinical studies of NO activity in viral, bacterial, protozoal and fungal infections.

## Introduction

Nitric oxide (NO), an inorganic molecule, is generated endogenously by prokaryotes and eukaryotes from L-arginine by a family of NO synthase enzymes (NOS; Table 1.1). ^1^ In higher animals, it is also generated by reduction of dietary and endogenous nitrate (NO _3_^−^) to nitrite (NO _2_^−^) and thence NO ( Table 1.2). NO is a pleiotropic signalling molecule involved in vascular, neuronal and metabolic regulation and has multiple physiological effects including lowering blood pressure, increasing exercise performance, and reversing metabolic syndrome. Underlying these processes, NO modulates multiple cell types including leucocytes, ^2^ platelets, ^3^ endothelial cells and smooth muscle cells, and neuronal, cardiac and renal function. Three isoforms of NOS exist in eukaryotes: neuronal (nNOS, NOS1), inducible (iNOS, NOS2) and endothelial (eNOS, NOS3). In multicellular organisms, NOS1-3 produces NO that broadly mediates neurotransmission, cyto-toxicity and vascular regulation respectively. Within cells, NO interacts with mitochondrial respiration, activates metabolic regulatory pathways and reduces oxidative stress.

**Table 1.** Chemical equations relevant to the nitric oxide system.

No.	Substrate	Product	Enzyme	Biochemical equation
1.	L-arginine	NO	Nitric oxide synthase	2 L-arginine + 3 NADPH + 3 H ^+^ + 4 O _2_ ➔ 2 L-citrulline + 2 NO + 3 NADP ^+^ + 4 H _2_O
2.	Dietary nitrate	NO		NO _3_^−^ + e + 2 H ^+^ ➔ H _2_O + NO _2_^−^ 2 NO _2_^−^ + 2 H ^+^ ➔ 2 HNO _2_ ➔ N _2_O _3_ + H _2_O N _2_O _3_ ➔ NO + NO _2_
3.	NO	Peroxynitrite		NO ^•^ + O _2_^•−^ ➔ OONO ^−^
4.	Superoxide	Hydrogen peroxide	Superoxide dismutase	O _2_^−^ ➔ O _2_ + H _2_O _2_
5.	Nitrite	Peroxynitrous acid		NO _2_^−^ + H _2_O _2_ ➔ ONOOH
6.	NO	Nitrogen dioxide		2 NO + O _2_ ➔ 2 NO _2_; ONOOH ➔ NO _2_ + OH
7.	NO	Dinitrogen trioxide		NO + NO _2_ ➔ N _2_O _3_
8.	Nitrogen dioxide	Dinitrogen tetroxide (amyl)		2 NO _2_ ➔ N _2_O _4_
9.	Hydrogen peroxide	Hydroxyl radicals		H _2_O _2_ ➔ OH ^−^ + OH ^•^ ➔ DNA damage
10.	NO	Nitrous oxide	NO reductase	2 NO + NAD(P) H + H ^+^ ➔ N _2_O + H _2_O + NAD(P) ^+^
11.	NO	Nitrate	NO dioxygenase	Fe ^3+^(O _2_^−^) + NO ➔ Fe ^3+^ + NO _3_^−^

Note: Many of these reactions can occur in the opposite direction, *e.g.* inhaled NO _2_, an environmental pollutant, is a source of bioactive intravascular nitrite. ^187^

As people age, endothelial-derived vascular NO levels fall and so vascular function declines causing relative endothelial dysfunction, pro-platelet and pro-inflammatory effects, and increased smooth muscle proliferation. Vascular NO levels are even lower in people with established vascular disease, *e.g.* those with stroke. ^4^^,^^5^ Numerous viruses (including adeno, Coxsackie, coronavirus, cytomegalovirus, echovirus, herpes simplex, human T-cell leukaemia virus type-1, human immunodeficiency virus, influenza, measles, mumps, polio) and bacteria ( *Leptospira* spp.) can infect ^6^^–^^8^ and damage endothelium and so further reduce vascular NO levels. This also appears to occur in SARS-CoV-2 infection. ^9^

Most physiological effects of NO are modulated by cyclic guanosine monophosphate (cGMP, second messenger), ^10^^,^^11^ and terminated when cGMP is metabolised by phosphodiesterase-5 (PDE5). This combined L-arginine/nitrate-NO-cGMP-PDE5 system (or nitric oxide system) comprises one of two key vasculo-protective pathways, the other being the prostaglandin-cyclic adenosine monophosphate-phosphodiesterase-3 pathway (PG-cAMP-PDE3, or prostacyclin system, see below).

The NO system may be enhanced or stimulated exogenously with substrate (L-arginine, organic nitrate, inorganic nitrite or nitrate), NO gas, and PDE5 inhibitors. These can be inhaled or administered *via* transdermal, sublingual, oral, intranasal or intravenous routes (see below). Since endogenous NO generated by inducible NOS plays a key role in defence against multiple microbial pathogens (including viruses, bacteria, protozoa and fungi/yeast), this raises the possibility that exogenous NO might have therapeutic potential as a broad-spectrum antimicrobial, and this is the topic of this review.

## Methods for the review

There are numerous publications in this research area and our intention was not to perform a systematic review of these; rather we present exemplars from the research field. We identified publications relating to the effect of NO on viruses, bacteria, protozoa and fungi/yeasts from searches of our own reference libraries, PubMed and Google, and reference lists given in earlier reviews and commentaries. ^12^^–^^20^

The primary searches for relevant studies for inclusion were done through PubMed from inception to 4 May 2021, in English with the following disease terms: “microbe” or “virus” or “bacteria” or “protozoa” or “fungi” or “yeast”. The results of these searches were crossed with the drug terms “nitric oxide” or “nitrite” or “nitrate”, and the identified abstracts were screened by one or more researchers. Earlier studies, including published reviews, were also identified from the files of the senior author. Studies included *in vitro*, *in vivo* and clinical trials. Although other drug classes, such as statins and angiotensin-converting enzyme inhibitors, enhance endothelial production of nitric oxide, their main effects are mediated through other pathways, and we have not assessed them here even though they may attenuate microbial disease severity.

## Chemistry and biology of nitric oxide

NO is a small diatomic hydrophobic colourless gas that diffuses easily and has a short half-life. With an unpaired electron (NO ^•^), it is a free radical and so is chemically reactive and unstable in the presence of oxygen and superoxide producing reactive nitrogen oxide species (RNOS). In reality, the chemistry of NO is more complex and it exists in several redox forms: nitrosonium cation (NO ^+^), NO (NO ^•^) and nitroxyl anion NO ^−^). ^21^

As an inorganic molecule, its central role in biology as a signalling molecule was only discovered in the 1980s, ^22^ the identification of which led to the Nobel Prize for Physiology and Medicine in 1998. ^23^ Ironically, the medicinal use of NO in the form of glyceryl trinitrate (GTN) for angina prophylaxis antedates the modern understanding of the biological synthesis and role of NO by more than a century. ^24^

## Experimental studies demonstrating nitric oxide inhibition of …

### Viruses

Numerous preclinical *in vitro* studies have demonstrated that NO sources (stimulated endogenous NO, inorganic and organic nitrates, L-arginine) and PDE5-inhibitors can reduce replication in all seven types of virus as defined in the Baltimore classification ( Table 2); ^25^ this includes Class IV viruses (positive-sense RNA viruses) incorporating several coronaviruses ^26^^–^^31^ including SARS-CoV-2. ^32^^–^^34^ Most studies showed efficacy although neutral studies were reported for porcine reproductive and respiratory virus (an arterivirus, which is closely related to coronaviruses) and rhinovirus. ^35^^,^^36^

**Table 2.** Studies assessing the effect of nitric oxide on selected viruses ordered by Baltimore class. ^25^

Year	Virus (Baltimore class)	Disease (human unless stated)	NO source	*In vitro* cell lines	*In vivo*	Results
	**dsDNA (I)**					
2020 ^43^	Cytomegalovirus	In compromised immune systems	DETA/NO	MRC-5, ARPE-19		Reduced replication
1993 ^188^	Ectromelia	Mousepox	SNAP	293	C57BL/6NCR mice	Reduced replication
1994 ^189^	Epstein-Barr	Infectious mononucleosis, Burkitt lymphoma	Constitutive lymphocyte	Human B-lymphocytes		Reduced reactivation
1993 ^188^	Herpes simplex-1	Cold sores	SNAP	293	C57BL/6NCR mice	Reduced replication
1993 ^190^	Herpes simplex-1	Cold sores	SNAP	RAW 264.7		Reduced replication
2015 ^126^	Human papilloma virus	Anogenital warts	Acidified NaNO _2_		Human	Cure in 31% (active) vs 14% (control)
1999 ^122^	Molluscipoxvirus	Molluscum contagiosum	Acidified NaNO _2_		Human	Cure in 75% (active) vs 21% (control)
1993 ^188^	Vaccinia	Human “pox”	SNAP	293	C57BL/6NCR mice	Reduced replication
1995 ^191^	Vaccinia	Human “pox”	L-arg	RAW 264.7		Reduced replication
1995 ^192^	Vaccinia	Human “pox”	iNOS	BSC-40, HeLa G		Reduced replication
	**ssDNA (II)**					
2017 ^193^	Porcine circovirus-2	Swine multisystemic wasting syndrome	GSNO	PK-15	BALB/c mice	Reduced replication & infection
2009 ^194^	Porcine parvovirus	Swine embryonic/foetal death	SNAP, L-arg	PK-15		Reduced replication
	**dsRNA (III)**					
1996 ^195^	Avian (ortho-) reovirus	Avian arthritis, tenosynovitis	LPS-stimulated macrophages	HD11		Reduced replication
	**(+)ssRNA (IV)**					
1997 ^196^	Coxsackievirus (B3)	Pleurodynia, myocarditis, pericarditis, and hepatitis	iNOS transfection, SNAP	HeLa		Reduced replication, RNA and protein synthesis
2006 ^197^	Dengue virus-2	Viral haemorrhagic fever	SNAP	LLC-MK2 monkey kidney		Reduced RNA and protein synthesis
1999 ^198^	Human immunodeficiency virus	Acquired immune deficiency syndrome	SNAP	Human monocytes. U1 cells		Reduced replication in monocytes. Increased replication in U1 cells
1997 ^199^	Japanese encephalitis virus	Encephalitis	SNAP, IFN-γ activated macrophages	Murine RAW 264.7 and N18 cells		Reduced replication
2005 ^200^	Mengovirus	Acute fever	Dipyridamole	HeLa or L cells		Reduced replication
1997 ^26^	Murine coronavirus	Murine hepatitis	SNAP	OBL21a		Reduced replication
1998	Poliovirus	Poliomyelitis	GTN	HeLa, U937		Reduced replication
2010 ^35^	Porcine reproductive & respiratory virus	Swine reproductive failure, respiratory tract infection	SNAP, NAP	Marc-145 cells		NAP (but not SNAP) inhibited replication
2010 ^27^	Porcine respiratory coronavirus	Swine reproductive failure, respiratory tract infection	SNAP	STC		Reduced replication
2020 ^30^	OC43 coronavirus	Cold	NO gas	HCT-8 cells		Reduced replication
1999 ^36^	Rhinovirus type 23	Common cold	SNAP, SNP, PAPA-NONOate	BEAS-2B cells, MRC-5 cells		No effect on replication
1996 ^201^	Sindbis	Sindbis fever. Murine encephalomyelitis	SNAP, SNP, NOS	N18	BALB/cJ, BALB/cByJ, scid/CB17 mice	Increased cell viability; less death
2004 ^28^	SARS-CoV-1	SARS	SNAP, SNP	Vero E6		SNAP (not SNP) reduced replication
2004 ^31^	SARS-CoV-1	SARS	NO gas		Human (n=14)	Improved arterial oxygenation, less lung radiological infiltrates
2005 ^29^	SARS-CoV-1	SARS	SNAP, iNOS	Vero E6		Reduced replication
2020 ^32^	SARS-CoV-2	COVID-19	SNAP	Vero E6		Reduced replication, recombinant protease activity
2020 ^33^	SARS-CoV-2	COVID-19	Dipyridamole	Vero E6	Human (n=31)	Reduced replication. Clinical improvement, increased Lϕ count
	**(-)ssRNA (V)**					
1982 ^202^	Influenza A 42/72, 1/79, A/fowl plague	‘Flu	Dipyridamole		White mice	Reduced replication. Infection prevention
1999 ^203^	Influenza A/B	‘Flu	SNAP, SNP, SIN-1	Mabin Darby		Reduced replication
2013 ^204^	Influenza H1N1, H3N2, B HongKong	‘Flu	NO gas	MDCK		Reduced infectivity. Inhibition of neuraminidase
2000 ^205^	Lymphocytic choriomeningitis virus	Meningoencephalitis	IFN-γ		HBV transgenic/iNOS knockout mice	NO mediates antiviral activity of IFN-γ
2006 ^206^	(Ortho)hantavirus	Haemorrhagic fever/pulmonary syndrome	SNAP, SIN-1	Vero E6	C57BL/6 (iNOS ^-/-^, ^+/+^) mice	Reduced replication
2006 ^207^	Parainfluenza virus	‘Cold’	DetaNONOate. SNAP. iNOS overexpression.	Cystic fibrosis epithelial cells		Reduced replication
2001 ^208^	Rabies virus	Rabies	SNP + ascorbate	Neuroblastoma cells		Reduced replication
1995 ^209^	Vesicular stomatitis	‘Flu. Bovine oral ulcers	SNAP	NB41A3		Reduced replication
	**ssRNA-RT (VI)**					
1995 ^210^	Friend leukaemia + spleen focus-forming	Murine leukaemia	SIN-1, SNP, SNAP	Dunni		Reduced replication (but not with NaNO _2_)
	**dsDNA-RT (VII)**					
2000 ^205^	Hepatitis B	Viral hepatitis	IFN-γ		HBV transgenic/iNOS knockout mice	NO mediates antiviral activity of IFN-γ

DETA/NO; diethylenetriamine NONOate; dsDNA: double-strand DNA (type I); dsDNA-RT: single-strand DNA-retro (type VII); dsRNA: double-strand RNA (type III); GSNO: S-nitrosoglutathione; IFN-γ: interferon-gamma; iNOS: inducible nitric oxide synthase; L-arg: L-arginine; Lϕ: lymphocyte; NaNO _2_: sodium nitrite; NAP: N-acetylpenicillamine; NO: nitric oxide; SARS: severe acute respiratory syndrome; SIN-1: 3-morpholinosydnonimine; SNAP: S-nitroso-L-acetylpenicillamine; SNP: sodium nitroprusside; ssDNA: single-strand DNA (type II); (-)ssRNA: negative-sense single-strand RNA (type V); (+)ssRNA: positive-sense single-strand RNA (type IV); ssRNA-RT: single-strand RNA-retro (type VI).

### Bacteria

Multiple studies have assessed the effect of NO on bacteria and inhibitory effects have been seen across a wide range of gram negative, gram positive and acid-fast bacteria ( Table 3). NO sources included L-arginine, NO, nitrite, organic nitrates, and endogenously-generated NO. Multiple mechanisms for efficacy have been reported, as discussed below.

**Table 3.** Studies assessing the effect of nitric oxide on selected bacteria.

Year	Bacteria	Disease (human unless stated)	NO source	*In vitro*	*In vivo*	Results
	**Gracilicutes (gram negative)**					
2005 ^211^	*Acinetobacter baumanii*	ICU organisms	NO gas (200 ppm)	Bacterial cfu		No viable bacteria by 4.8 (±1.3) hr
1993 ^212^	*Brucella abortus*	Brucellosis	Activated macrophages (IFN-γ)	BALB-c murine J774A.1		Reduced cfu
2003 ^186^	*Burkholderia pseudomallei*	Melioidosis	Activated macrophages (IFN-β)	RAW 264.7 murine macrophages		Reduced intracellular bacteria
1993 ^213^	*Chlamydia trachomatis*	Trachoma, pelvic inflammatory disease	Activated McCoy cells (IFN-γ)	Murine fibroblasts		Reduced infectivity
1992 ^214^	*Ehrlichia*	Ehrilichiosis	Activated macrophages (L-arginine/IFN-γ). SNP	Murine macrophages		No viable bacteria. Dependent on iron (not cGMP)
2005 ^211^	*Enterobacter aerogenes*	ICU organisms	NO gas (200 ppm)	Bacterial cfu		No viable bacteria by 4.8 (±1.3) hr
2005 ^211^	*Escherichia coli*	ICU organisms	NO gas (200 ppm)	Bacterial cfu		No viable bacteria by 4.8 (±1.3) hr
1992 ^215^	*Francisella tularensis*	Tularaemia	Activated macrophages (L-arginine/IFN-γ)	Murine macrophages		Suppressed growth
1998 ^118^	*Helicobacter pylori*	Gastritis, gastric/duodenal ulcers	Acidified (pH 2) potassium nitrite	Bacterial cfu		No viable bacteria at KNO _2_ >500 μmol/L
2005 ^211^	*Klebsiella pneumoniae*	ICU organisms	NO gas (200 ppm)	Bacterial cfu		No viable bacteria by 4.8 (±1.3) hr
1992 ^216^	*Legionella*	Legionnaires/Pontiac fever	Activated macrophages (IFN-γ)	RAW 264.7/HL-60		Few viable bacteria
2005 ^211^	*Pseudomonas aeruginosa*	ICU organisms	NO gas (200 ppm)	Bacterial cfu		No viable bacteria by 4.8 (±1.3) hr
1993 ^217^	*Rickettsia*	Spotted fever, typhus	Activated macrophages (IFN-γ/TNF-α)	Murine fibroblasts		Reduced infection
1995 ^38^	*Salmonella enterica (Typhimurium)*	Typhoid fever	SIN-1, GSNO, (diethylenetriamine-NO)	Suspension		SIN-1: oxygen-dependent cytostasis. GSNO: oxygen-independent cytostasis
2005 ^211^	Serratia marcescens	ICU organisms	NO gas (200 ppm)	Bacterial cfu		No viable bacteria by 4.8 (±1.3) hr
2005 ^211^	Stenotrophomonas maltophilia	ICU organisms	NO gas (200 ppm)	Bacterial cfu		No viable bacteria by 4.8 (±1.3) hr
1992 de Giusti ^218^	Yersinia pestis	Plague (bubonic, pneumonic, septicaemic)	NaNO _2_, NaNO _3_. KNO _3_	Pork meat		Reduced growth
	**Firmicutes (gram positive)**					
1981 ^219^	*Bacillus cereus*	Gastroenteritis	Nitrosothiols (RSN=O)	Suspension of spores		Inhibition of spore germination
1976 ^44^	*Clostridium perfringens*	Gastroenteritis	Sodium nitrite			Reduced cfu, G _AP-DH_ and aldolase _activity, and_ free sulfhydryl groups
1994 ^220^	*Listeria*	Listeriosis	Sublethal inoculum		C57BL/6 mice	L-NMMA inhibition of NO worsened outcome
2005 ^211^	*Staphylococcus aureus*	ICU organisms	NO gas (200 ppm)	Bacterial cfu		No viable bacteria by 4.8 (±1.3) hr.
2012 ^221^	*Staphylococcus aureus*	Wound infection	Probiotic NO gas patch		Ischaemic/infected ( *S. aureus*) full thickness wounds in rabbits	Improved closure.
2013 ^147^	*Staphylococcus aureus*	Experimental biofilms	Glyceryl trinitrate	Biofilm		Antimicrobial synergisation with citrate and ethanol
2018 ^148^	*Staphylococcus aureus*	Experimental biofilms	Isosorbide mononitrate	Biofilm		Increased dispersal (conversion of sessile to planktonic cells)
2005 ^211^	*Streptococci (group B)*	ICU organisms	NO gas (200 ppm)	Bacterial cfu		No viable bacteria by 4.8 (±1.3) hr.
	**Acid fast**					
1991 ^222^	*Mycobacterium avium*	Atypical respiratory TB	Activated macrophages (TNF)	Human macrophages		Reduced growth
1991 ^223^	*Mycobacterium leprae*	Leprosy	Activated macrophages (IFN-γ)	Murine macrophages		Reduced *M. leprae* metabolism
2003 ^48^	*Mycobacterium tuberculosis*	Respiratory TB	Adjuvant L-arginine		Smear positive TB	Improved outcome (weight, less cough)
2004 ^224^	*Mycobacterium ulcerans*	Buruli skin ulcer	Acidified nitrite (40 mM)	In suspension		Bacteriocidal

cfu: colony forming units; GSNO: S-nitrosoglutathione; IFN-γ: interferon-gamma; MRSA: methicillin resistant S. aureus; ppm: parts per million; SIN-1: 3-morpholinosydnonimine; TB: tuberculosis; TNF: tumour necrosis factor.

### Protozoa

NO sources have been tested on both intracellular and extracellular protozoa ( Table 4) with sources involving activated macrophages, sodium nitrite, glyceryl trinitrate, sodium nitroprusside (SNP) and S-nitroso-L-acetylpenicillamine (SNAP).

**Table 4.** Studies assessing the effect of nitric oxide on a non-inclusive list of protozoa.

Year	Protozoa	Disease (human unless stated)	NO source	*In vitro*	*In vivo*	Results
	**Extracellular**					
1992 ^225^	*Entamoeba histolytica*	Amoebiasis	Activated macrophages (IFN-γ/LPS)	Murine macrophages		Reduced infection
1992 ^226^	*Naegleria fowleri*	Meningitis	BCG-activated macrophages	Female C57BL/6 mice		Destruction of amoebae
1994 ^227^	*Opisthorchis*	Opisthorchiasis, cholangiocarcinoma				
2013 ^228^	*Plasmodium berghei*	Murine cerebral malaria	TD GTN		Prevention, and adjunctive treatment	Reduced infection, and improved outcome
1989 ^229^	*Schistosoma mansoni*	S chistosomiasis, intestinal	Activated macro-phages (TNF)	Female C57BL/6 mice		Larval cytotoxicity
2017 ^230^	*Schistosoma japonicum*	S chistosomiasis, intestinal	Endogenous from iNOS		Sprague-Dawley rats	Reduced granuloma formation
	**Intracellular**					
2000 ^123^	*Leishmania major/tropica*	Leishmaniasis	Acidified sodium nitrite	BALB/c mice macrophages	Human cutaneous *L. tropica*	Reduced amastigotes and promastigotes. 28% patients improved, 12% cured.
2000 ^185^	*Leishmania major*	Leishmaniasis	Activated macrophages (IFN-α/β)	CD1/C57BL/6 mice macrophages		Reduced intracellular parasites
2016 ^231^	*Leishmania* spp.	Leishmaniasis	SNP	BALB/c mice macrophages		Reduced amastigotes and promastigotes
1990 ^232^	*Toxoplasma gondii*	Toxoplasmosis	Activated macrophages (IFN-γ/LPS)	Murine macrophages		Reduced growth
1996 ^233^	*Toxoplasma gondii* (ME49)	Toxoplasmosis	Spleen cells		C57Bl/6 mice	Increased ocular inflammation with aminoguanidine
1992 ^169^	*Trypanosoma brucei gambiencei/brucei*	African trypanosomiasis (sleeping sickness)	Activated macrophages (BCG-infected mice or IFN-γ/LPS)	Murine macrophages	BCG-infected mice	No proliferation (cytostasis). Reduced parasitaemia/prolonged survival
1994 ^170^	*Trypanosoma brucei*	African trypanosomiasis (sleeping sickness)	SNAP. Activated macrophages (IFN-γ)	Murine peritoneal macrophages	C3H.He mice	*In vitro*: NO inhibits proliferation. *In vivo*: NO reduced protozoa T-cell proliferative responses

BCG: bacillus Calmette-Guerin; GTN: glyceryl trinitrate; IFN-γ: interferon-gamma; iNOS; inducible nitric oxide synthase; LPS: lipopolysaccharide; SNAP: S-nitroso-L-acetylpenicillamine; SNP: sodium nitroprusside; TD: transdermal; TNF: tumour necrosis factor.

### Fungi and yeasts

The effects of NO on several fungi and yeasts have been studied ( Table 5). NO was donated exogenously through stimulating macrophages or as acidified nitrite. *In vitro* experiments demonstrated reduced replication whilst *in vivo* experiments in mice showed reduced infection.

**Table 5.** Studies assessing the effect of nitric oxide on a non-inclusive list of fungi and yeasts.

Year	Fungi/Yeasts	Disease (human unless stated)	NO source	*In vitro*	*In vivo*	Results
	**Fungi**					
1999 ^234^	*Aspergillus fumigatus*	Aspergillosis	Activated macro-phages (IFN-γ)	Rat alveolar macrophages		Reduced infection
1998 ^121^	*Epidermophyton floccosum*	Tinea pedis	Acidified NaNO _2_		Human (n=35)	Cure in 81% (active) vs 31% (control)
1994 ^235^	*Histoplasma capsulatum*	Histoplasmosis (‘flu-like)	IFN-γ/LPS activated macrophages	C57BL/6 mice		Reduced infection
1999 ^236^	*Pneumocystis carinii*	Pneumonia	IFN-γ activated macrophages via L-arginine	Sprague Dawley rats		Killed *P. carinii*
1998 ^121^	*Trichophyton rubrum, T. interdigitale*	Tinea pedis	Acidified NaNO _2_		Human (n=35)	Cure in 81% (active) vs 31% (control)
	**Yeasts**					
1993 ^237^	*Candida albicans*	Candidiasis (oropharyngeal, vulvovaginal), candidaemia	Murine macrophages	*C. albicans* infection	Mice	Reduced infection
1991 ^238^	*Cryptococcus neoformans*	Cryptococcosis (pneumonia, meningitis, encephalitis)	Acidified NaNO _2_	*C. neoformans* culture		Reduced replication
1999 ^234^	*Cryptococcus neoformans*	Cryptococcosis	Activated macro-phages (IFN-γ)	Rat alveolar macrophages		Reduced infection
2018 ^239^	Dermantophytes	Onychomycosis, tinea pedis	NVN1000	Macrodilution broth test		78-99% kill

IFN-γ: interferon-gamma; LPS: lipopolysaccharide; NaNO _2_: sodium nitrite.

### Derivatives of nitric oxide

Whilst endogenous NO derived from eNOS and nNOS is physiologically active *via* its second messenger (cGMP), the antimicrobial effects of NO relate to its toxic effects when present at higher concentrations. Although it is technically challenging to measure free NO concentrations, studies suggest that NO concentrations derived from iNOS are 10–100× higher than those resulting from eNOS/nNOS ( Table 6). NO concentrations resulting from exogenous administration lie between those from eNOS/nNOS and iNOS but approximate more closely to those from iNOS than eNOS. Importantly, much antimicrobial NO activity is likely to reflect the effects of derivative molecules rather than NO itself:

• Nitric oxide (NO ^•^). In general, bacteria deficient in low molecular weight thiols such as glutathione ( *e.g. Staphylococci* spp.) are sensitive to attack by NO whereas those with high thiol concentrations are resistant to NO.
• Peroxynitrite (OONO ^-^, Table 1.3). The reaction between NO and superoxide means that NO synergises with the respiratory burst, another antimicrobial system present in phagocytic cells. Experimentally, this synergism can be inhibited with the addition of superoxide dismutase which converts superoxide into molecular oxygen and hydrogen peroxide ( Table 1.4).
• Peroxynitrous acid (ONOOH, Table 1.5), *e.g.* toxic to *Escherichia coli.* ^37^
• Nitrogen dioxide (NO _2_, Table 1.6), *e.g.* toxic to *E. coli.* ^37^
• Dinitrogen trioxide (N _2_O _3_, Table 1.7).
• Dinitrogen tetroxide (N _2_O _4_, Table 1.8).
• S-nitrosothiols (RSNO, *e.g.* S-nitrosoglutathione), *e.g.* toxic to *E. coli* and *Salmonella enterica* serovar typhimurium. ^38^ RSNO reacts with protein sulphydryl groups changing their function. Thiol concentrations do not appear to determine sensitivity to peroxynitrite and S-nitrosothiols.
• Dinitrosyl-iron ((2 RS)-Fe-(2 NO)). The reaction of NO with iron or iron–sulphur molecules can: inactivate enzymes such as aconitase (which converts citrate to isocitrate in the citric acid cycle), ribonucleotide reductase and ubiquinone reductase; increase free ferrous (Fe ^2+^) which causes oxidative damage; and deplete iron stores.
• RNOS (especially auto-oxidised products of NO).

**Table 6.** Nitric oxide concentrations following endogenous synthesis by nitric oxide synthase, and exogenous NO donors.

NO	eNOS/nNOS	iNOS	Exogenous NO
Source	Endothelium, neurones	Intracellular, e.g. macrophages	Exogenous
Role	Cell signalling	Microbial killing	Vasodilation, antiplatelet
Synthesis	Constitutive, intermittent (“dripping tap” ^17^), calcium-dependent, feedback controlled	Inducible, continuous (“fire hose” ^17^), calcium-independent/cytokine-microbial dependent. Part of innate immunity	
Concentration	0.1-5 nM ^240^	>10 μM ^240^	SNP, 52 nM ^241^
Targets	sGC (CcOX)	Aconitase, NADH dehydrogenase, succinate dehydrogenase, metalloenzymes, ribonucleotide reductase, DNA	sGC
Effects	Reversible	Irreversible. Nitrosation, nitration, oxidation.	Reversible

CcOX: cytochrome c oxidase (Complex IV); sGC: soluble guanylate cyclase; SNP: sodium nitroprusside.

Since these molecules differ in their stability, reactivity, location and cellular diffusivity, the overall effect of NO will depend on the molecular species involved and its location.

### Cellular and other targets of nitric oxide

The targets for NO and associated reactive nitrogen species are multitudinous:

• DNA, through deamination of adenine, cytosine and guanine; ^39^ cross-linking; breakage of strands; inhibition of DNA repair enzymes such as DNA alkyl transferases (and so preventing transfer of the guanine alkyl group to protein); and disruption of DNA replication by inhibition of ribonucleotide reductase; ^40^ as in *S. enterica* and vaccinia virus.
• RNA, through disruption of RNA replication by inhibition of viral ribonucleotide reductase.
• Inhibition of mitochondrial function, specifically through inactivation of iron-sulphur complexes within respiratory chain enzymes. ^41^
• Protein modification at cysteine, methionine, phenylalanine, tryptophan and tyrosine residues, *e.g.* by RNOS. Such protein effects will reduce enzyme activity, as seen for DNA, proteases ^42^ and mitochondrial function, as in Coxsackievirus. ^42^
• Limit late protein synthesis, *e.g.* through posttranslational modification of viral proteases. (Early protein translation/synthesis is not typically affected.)
• Induction of lipid peroxidation.
• Limit glutaminolysis by shuttling glutamine to glutathione synthesis, as in cytomegalovirus. ^43^
• Interaction with sulfhydryl-containing constituents of the bacterial cell. ^44^
• Disrupt zinc homeostasis, as in *S. enterica.* ^45^
• Limit virion assembly/particle formation.
• Reduce bacterial adhesion to NO-releasing surfaces. ^46^

Nitric oxide may also play an augmenting role as an antimicrobial agent. Examples include the adjuvant roles of NO when given with type I interferons in the treatment of DNA viruses ^47^ and L-arginine when given with conventional chemotherapy in smear-positive TB. ^48^

In addition, NO’s vasculo-active effects are likely to be beneficial in preventing infection and its severity, with NO:

• Reversing endothelial dysfunction and so potentially reducing endotheliitis, ^9^ as occurs in COVID-19. ^7^^,^^8^
• Reducing leucocyte function ( *e.g.* adhesion, chemotaxis, phagocytosis); ^2^ COVID-19 is associated with increased phagocyte counts. ^8^^,^^49^
• Reducing platelet activation and platelet–leucocyte conjugation and so reducing micro- and macro-thrombosis, as seen in COVID-19. ^8^^,^^50^
• Improving organ blood flow and perfusion through smooth muscle relaxation and vasodilation and so likely reversing infection-related vasoconstriction as seen in COVID-19, ^8^ including in the pulmonary circulation. ^51^

These actions of NO are all mediated *via* the second messenger cGMP.

### Antimicrobial production of nitric oxide

NO is produced by some bacteria, archaea and yeasts *via* several pathways including denitrification of nitrate to nitrite and then to NO ^52^ and oxidation of L-arginine to NO and L-citrulline as catalysed by a bacterial nitric oxide synthase (bNOS), a process that can be inhibited by NOS inhibitors. ^53^ Whilst eukaryotic NOS contains both catalytic and reductase domains, prokaryotic bNOS lacks the latter relying instead on other cellular reductases to generate NO; the one exception to this is the bNOS present in *Sorangium cellulosum* which does include a reductase module.

In contrast to the signalling role of NO in mammals, NO synthesis in bacteria has multiple functions which vary between antimicrobial species: ^54^^–^^57^

• Protection against oxidative stress with NO limiting thiol reduction and so the formation of hydroxyl radicals ( *Bacillus anthracis/subtilis, Staphylococcus aureus* ^54^) ( Table 1.9).
• Protection against oxidative stress with NO activating catalase ( *B. subtilis*). Such defence will limit damage from phagocytic respiratory bursts. ^54^
• Protection against oxidative stress by reducing endogenous NO synthesis and increasing the expression of NO dioxygenase to detoxify NO ( *Candida albicans*). ^56^
• Biosynthesis of toxins, *e.g.* thaxtomins (a phytotoxin) interfere with potato plant wall synthesis ( *Streptomyces turgidiscabies*). ^54^
• Activation of aerobic and nitrate respiration to optimise growth ( *S. aureus*). ^57^^,^^58^
• Protection against antimicrobial agents including amoxycillin, cefuroxime, gentamicin and novobiocin ( *B. anthracis/cereus/thuringiensis/weihenstephanensis*, *S. aureus*), ^55^ and azoles ( *Candida albicans*). ^56^

The production by some microbes of endogenous NO to protect against oxidative stress is ironic since hosts are using NO to try to destroy the microbe!

### Resistance to nitric oxide

Microbial resistance to antibiotics is an increasingly common problem and has left some bacteria with few treatment options, *e.g.* drug-resistant *Neisseria gonorrhoeae.* Hence, it is vital to consider whether resistance to NO is innate in some microbes or can be acquired. As already highlighted, some microbes have an intrinsic ability to produce their own NO and so attenuate the effects of oxidative stress ( *e.g. B. anthracis/subtilis, C. albicans*, *S. aureus* ^54,56^), activate aerobic respiration ( *S. aureus* ^57^) or protect against antimicrobial agents ( *B. anthracis/cereus/thuringiensis/weihenstephanensis*, *C. albicans*, *S. aureus* ^55,56^).

Microbes may also have mechanisms for deactivating NO. One mechanism is *via* a NO reductase which reduces NO to nitrous oxide and then nitrogen, *e.g.* as occurs in fungi ^59^ ( Table 1.10). Bacteria have different NO reductases but similarly produce nitrous oxide, ^59^ as seen in *Pseudomonas aeruginosa.* ^52^ Loss-of-function mutations in NO reductase may be lethal, possibly because intracellular NO concentrations rise to toxic levels.

A second mechanism for detoxifying NO is *via* NO dioxygenase oxidation to nitrate ( Table 1.11). The pre-eminent NO dioxygenase is flavohaemoglobin, ^60^ as present in bacteria ( *e.g. Salmonella enterica*, *S. aureus*, *Vibrio cholerae*, *Yersinia pestis*, ^20^^,^^61^^,^^62^) and yeasts. A related haemoglobin, truncated haemoglobin, detoxifies NO in mycobacteria. Of note, *Mycobacterium leprae* has undergone reductive genome evolution losing more than 2,000 genes, including some that protect against RNOS; as a result, *M. leprae* has fewer defences against NO than *Mycobacterium tuberculosis.* ^63^ Bacterial lactate dehydrogenase also detoxifies NO, as seen in *S. aureus.* ^20^ Importantly, these detoxifying enzymes only cope with low levels of NO and are not protective against high NO levels.

As a result, microbes show differing sensitivities to NO, as seen for common airways pathogens where sensitivity was ranked (sensitivity most to least): *P. aeruginosa* ~ *C. albicans* > *S. aureus* > *Klebsiella pneumoniae* ~ *Staphylococcus epidermis.* ^64^

However, there is little evidence that bacteria can acquire *de novo* resistance to NO, as confirmed in experiments on strains of *E. coli*, *P. aeruginosa*, *S. aureus* and *Staphylococcus epidermidis.* ^65^ This property is unsurprising since NO has multiple mechanisms for antimicrobial activity and these are likely to be invoked orders of magnitude faster than any microbe can process metabolically, especially if protein synthesis is required. Equally, the main mechanisms for antibiotic resistance (drug inactivation, altered binding sites or metabolism and reduced drug permeability) are unlikely to be relevant to many NO sources. Whether viruses, protozoa and fungi can develop resistance to NO remains unclear.

## Administering nitric oxide, donors and related compounds

### L-arginine

In the presence of NOS, administration of L-arginine may enhance NO synthesis ( Table 7) although intracellular L-arginine levels are not normally rate limiting and so administration may not have physiological effects. ^66^ Although oral preparations of L-arginine are commercially available, consumption of high doses is associated with profuse diarrhoea (P Bath, personal observation).

**Table 7.** Nitric oxide sources.

Intervention	Example	Administration	Licensed for use in (BNF)	Antimicrobial effects: target (disease)
L-arginine	Dietary: meat	Oral	N/A	None reported
	Powder		N/A	Mycobacteria tuberculosis (pulmonary tuberculosis) ^48^
	Liquid	Intravenous	N/A	None reported
Inorganic nitrite	Acidified sodium nitrite (NaNO _3_) cream	Topical		*Burkholderia cepacia*, dermatophytes (tinea pedis), ^121^ pox virus (molluscum contagiosum), ^122^ Leishmaniasis, ^123^ *Mycobacterium ulcerans* (Buruli ulcer), ^124^ human papilloma virus (anogenital warts), ^126^ *Propionibacterium acnes*, *P. aeruginosa*, *S. aureus* (including MRSA ^125^). ^105^
		Oral	N/A	Food preservation: ^145^ *Clostridium botulinum*
		Intravenous	Cyanide poisoning (given with sodium thiosulfate) ^145^	None reported
Inorganic nitrate	Silver nitrate	Topical	Common wart (human papilloma virus)	Human papilloma virus (Common wart). ^127^
	Dietary: beetroot, celery, rocket, spinach	Oral	N/A	*Lactobacillus*, *Streptococcus* (tooth decay), *C. albicans* (oral infection), *Campylobacter*, *E. coli* 0157, *Salmonella enterica* (Typhimurium), *Shigella sonnei*, *Yersinia enterocolitica* (gastroenteritis) ^113^
Organic nitrate	Glyceryl trinitrate (GTN)	Topical patch	Prophylaxis of angina and phlebitis	Malaria (murine cerebral malaria). ^228^ Non-specific infections (presenting as stroke mimics). ^150^
		Ointment	Treatment of anal fissure	None reported
		Sublingual	Prophylaxis-treatment of angina	None reported
		Intravenous	Hypertension/myocardial ischaemia after cardiac surgery. Congestive heart failure. Unstable angina	*E.coli*, *P. Aeruginosa*, *S. aureus* in solution. ^242^ *S. aureus*, MRSE, *P. aeruginosa*, *C. albicans* in biofilms ^147^
	Isosorbide dinitrate (ISDN)	Oral	Prophylaxis/treatment of angina. Left ventricular failure	None reported
		Sublingual	Prophylaxis/treatment of angina	None reported
		Intravenous	Prophylaxis/treatment of angina. Left ventricular failure	None reported
	Isosorbide mononitrate (ISMN)	Oral	Prophylaxis of angina. Adjunct in congestive heart failure.	*S. aureus* in biofilms ^148^
Spontaneous nitric oxide donors	Sodium nitroprusside (SNP)	Intravenous	Hypertensive emergencies. Controlled hypotension. Acute/chronic heart failure	*E.coli*, *P. Aeruginosa*, *S. aureus* in solution. ^242^ *Bacillus licheniformis, Candida albicans, Escherichia coli* BW20767, *Fusobacterium nucleatum, Serratia marcescens* MG1, *S. epidermidis, Vibrio cholerae* 92A1552. ^243^ *Leishmania* spp. ^231^
Nitric oxide	Nitric oxide (NO) gas	Gas	(Neonatal pulmonary hypertension)	*E. coli*, *P. aeruginosa*, *S. aureus.* ^244^ SARS-CoV-2 (ongoing trials: NCT04290871, NCT04305457, NCT04312243).
		Probiotic patch	N/A	*E. coli*, *S. aureus*, MRSA, *P. aeruginosa*, *T. mentagrophytes*, *T. rubrum* . ^221^^,^^245^
		NO releasing solution	N/A (in development)	*Propionibacterium acnes, T. mentagrophytes*, *T. rubrum.* SARS-CoV-2 (ongoing trial: NCT04337918)
PDE5 inhibitor	Dipyridamole	Oral	Post-stroke prophylaxis.	Picornaviridae, Togaviridae, Orthomyxoviridae, Paramyxoviridae, Herpetoviridae and Poxviridae. ^246^ Mengovirus. ^200^ SARS-CoV-2 (COVID-19 clinical trial). ^33^
	Sildenafil	Oral	Erectile dysfunction, pulmonary arterial hypertension	Adenovirus, Chikungunya, Cytomegalovirus, Dengue, Enterovirus 71, Influenza virus, Measles, Mumps, Rabies, Respiratory syncytial virus, Rubella, West Nile, Yellow Fever; Methicillin-resistant *Staphylococcus epidermidis.* ^247^^,^^248^ SARS-CoV-2 (ongoing trial: NCT04304313).

BNF: British National Formulary; N/A: not applicable.

### Inhaled nitric oxide

Gaseous NO may be inhaled with the aim of improving pulmonary haemodynamics and killing microbes. Multiple trials are underway for COVID-19 prevention and treatment ( Table 9). NO may also be created in real time by combining sodium nitrite and citric acid and administering this either as a nasal spray (for local therapy) or *via* nebuliser (for combined nasal and bronchial therapy).

### Organic nitrates

Organic nitrates such as GTN, isosorbide dinitrate (ISDN) and isosorbide mononitrate (ISMN) are widely used in vascular medicine for the prevention and treatment of angina, treatment of chest pain in unstable angina and myocardial infarction, treatment of severe heart failure, and blood pressure lowering after cardiac surgery and in acute stroke ( Table 7). There is increasing concern that chronic use of organic nitrates may cause major adverse cardiac events and death, ^67^ reduce daily activity, ^68^ and not improve quality of life or exercise capacity. ^68^ Potential explanations include the development of tolerance, and induction of endothelial dysfunction and cell damage through oxidative stress, *e.g.* production of free radicals/peroxynitrite. ^69^^,^^70^

Importantly, organic nitrates only release NO in cells and tissues expressing mitochondrial aldehyde dehydrogenase-2. ^71^ For example, SNP and SIN-1 inhibit monocyte chemotaxis whilst organic nitrates (ISDN, GTN and molsidomine) do not; ^72^ this contrasts with smooth muscle cells which vasodilate with all five agents. Since aldehyde dehydrogenase-2 suffers from use-inactivation, nitrate tolerance (tachyphylaxis) and endothelial dysfunction develops ^70^ and bioconversion only restarts following a nitrate-free period. Several *in vitro* studies have demonstrated the potential antimicrobial effects of organic nitrates ( Table 7). Other non-organic nitrates include pentaerythritol tetranitrate and erythrityl tetranitrate.

### Therapeutic inorganic nitrite and nitrate

Therapeutic use of inorganic nitrite is limited with intravenous administration used in cyanide poisoning ( British National Formulary). Topical acidified sodium nitrite has been shown to reduce cutaneous infections secondary to a variety of viruses and bacteria although its general use is probably limited by skin irritation and erythema ( Table 7).

### Dietary inorganic nitrate

NO may also be produced from dietary inorganic nitrate, as is present in high concentrations in green leafy and some root vegetables, *e.g.* spinach, lettuce, rocket, beetroot, celery, fennel, radish and Chinese cabbage. ^73^ Nitrate is absorbed from the proximal gastrointestinal tract, excreted by salivary glands, reduced to nitrite by oral bacteria and then absorbed in the gastrointestinal tract. A number of bacterial species situated on the dorsal surface of the tongue perform this conversion *via* nitrate reductases. ^74^ (In the absence of oxygen, nitrate and nitrite are commonly used by bacteria as terminal electron acceptors for respiration. ^75^) Through this symbiotic relationship, the mammalian host provides the nutrients and the environment in return for nitrite production by bacteria. ^76^

Absorbed and circulating nitrite is then further reduced to NO, a process that is enhanced in hypoxic or acidic conditions and by multiple mechanisms including deoxyhaemoglobin, deoxymyoglobin, xanthine oxidoreductase and endothelial nitric oxide synthase. ^77^^,^^78^ As such, most effects of dietary nitrate will be vascular and perivascular. The beneficial vascular protective effects of vegetable consumption are very clear epidemiologically, as present in the classical Japanese diet, ^79^ the Dietary Approaches to Stop Hypertension (DASH) diet, ^80^ and the Mediterranean diet. ^81^^,^^82^ Further, vegetable-derived nitrate may reduce the risk of gastrointestinal cancer. ^73^^,^^83^^,^^84^ The benefit on cancer is at variance with oral consumption of nitrite. Although nitrite is not carcinogenic *per se*, the processing and cooking of nitrite-cured meat can form carcinogens such as N-nitroso compounds and heterocyclic aromatic amines. In contrast, carcinogens are not formed when eating raw vegetable-derived nitrate. A recent meta-analysis showed an increased risk gastric cancer with oral nitrite but reduced risk with oral nitrate. ^85^ Dietary nitrate is known to modify the oral and gastric biome ( Table 7).

High dietary intake of nitrate is associated with many mechanisms that may have beneficial vascular, and potentially, antimicrobial effects. Experimentally, beetroot juice is often used as a potent source of dietary nitrate since dosing can be controlled and a nitrate-free placebo version is available for use in randomised controlled trials. Studies have shown that beetroot juice increases plasma nitrate and nitrite concentrations, ^86^^,^^87^ that most vascular effects are mediated *via* the second messenger cGMP, ^87^ tolerance does not develop (unlike with organic nitrates) ^73^ and inorganic nitrate does not lead to free radical formation. In clinical studies, beetroot juice has been given over weeks and months ^86^^–^^91^ and has been shown to have multiple effects with improved exercise performance (hence use by elite athletes) ^92^; improved cognitive performance in older people ^92^; vasodilation with reduced blood pressure ^89^^,^^90^^,^^92^^–^^95^; antiplatelet and anti-leucocyte effects and reduced platelet-leucocyte conjugation ^86^^,^^89^; improved endothelial function; reduced left ventricular volume; ^91^ improved metabolic profile; and improved oral health. ^89^ Beyond anti-inflammatory effects on blood cells, nitrite or nitrate reduce soluble pro-inflammatory factors including C-reactive protein, chemokine (C-X-C motif) ligand-1/2, endothelin-1, interleukins-1β/6/10/12p70, interferon-γ, monocyte chemoattractant protein and tissue necrosis factor-α. ^96^ Dietary nitrate has profound metabolic effects and appears to have the potential for reversing the metabolic syndrome and have anti-diabetic effects. ^97^ Overall, the pharmacological effects of beetroot juice have been demonstrated in younger and older people, and in people with cardiovascular disease, *e.g.* diabetes, obesity, hypertension, hypercholesterolaemia, heart failure and stroke. ^98^ Importantly, inorganic nitrate (given as beetroot juice) may be taken by pregnant women. ^99^ Experimentally, watermelon juice and chard gel may be used as an alternative source of dietary nitrate. ^100^^,^^101^

### Phosphodiesterase-5 inhibitors

PDE5-inhibitors, such as dipyridamole and sildenafil, enhance the physiological effects of NO as mediated by cGMP. Whether these agents should have antimicrobial effects is unclear since they do not enhance NO levels *per se*; nevertheless, both drugs have exhibited antimicrobial activity ( Table 7) and are being tested in COVID-19 trials ( Table 9). ^102^^,^^103^

### Stimulation of endogenous nitric oxide-dependent nitric oxide production

Endogenous NO production may also be stimulated externally. First, nasal breathing promotes the production of NO from the paranasal sinuses and this has bronchodilatory, vasodilatory and potential antimicrobial activities. ^104^ This natural defence mechanism may be attenuated with mouth breathing, as occurs with increasing age and obesity. Second, ultraviolet radiation (UVA and UVB) stimulates the release of NO from both keratinocytes and melanocytes; NO has multiple effects including attenuation of free radical damage, melanogenesis, blood pressure lowering ^105^ and potentially protection against skin infections.

### Novel nitric oxide agents

Recent research has focussed on the development of new antimicrobial NO delivery systems and some examples are listed:

• NO microspheres, *e.g.* biodegradable poly (lactic-co-glycolic acid) spheres loaded with S-nitroso-N-acetyl-D-penicillamine. ^106^
• NO-releasing nanoparticles, with activity against *Acinetobacter baumanii*, *C. albicans*, *Enterococcus faecalis*, *E. coli*, *K. pneumoniae*, *P. aeruginosa*, *S. aureus* (MRSA), *S. epidermidis*, *Trichophyton menatgrophytes.* ^105^^,^^107^
• Modified chitosan, *e.g.* against *Trypanosoma cruzi*, ^108^ *E. coli*, *S. aureus*, *Streptococcus mutans.* ^109^
• NO–metal complexes (zeolites), with activity against *B. subtilis*, *Clostridium difficile*, *E. coli*, *P. aeruginosa*, *S. aureus* (including MRSA). ^105^
• NONOoates (diazeniumdiolates), *e.g.* with activity against *C. albicans*, *E. coli.* ^105^^,^^110^
• NO coating of medical device surfaces and tubing, ^111^^,^^112^ *e.g.* using S-nitroso-N-acetyl-D-penicillamine to kill *Staphylococcus aureus* and *P. aeruginosa.*

NO sources can also be categorised by whether administration is local (e.g. cutaneous nitrite or intranasal preparations), systemic ( *e.g.* dietary nitrate or L-arginine, oral isosorbide or sildenafil, sublingual GTN, intravenous GTN or SNP) or mixed local and systemic (transdermal GTN). Local administration allows high and potentially cidal concentrations of NO to be achieved without unwanted systemic effects. Intravenous formulations might allow for systemic infections to be treated.

### Relevance of *in vitro* studies to preclinical and clinical studies

Most microbial studies presented above and in Tables 2-5 were performed *in vitro* and involved either inducing the L-arginine/NO pathway with cytokines ( *e.g.* interferon gamma [IFN-γ] and/or lipopolysaccharide [LPS]) or with NO sources (such as NO gas, nitrite, 3-morpholinosydnonimine, S-nitroso-L-acetylpenicillamine or sodium nitroprusside). However, the inhibitory effect of NO on microbes *in vitro* does not represent the complex biochemical environment that they face *in vivo* including the presence of NO derivatives such as peroxynitrite, microbial production of NO, microbial resistance to NO and excess NO synthesis. Nevertheless, there are many *ex vivo* and clinical examples where NO has been effective. These issues are now discussed.

## Nitric oxide for clinical infections

### Oral health and gastrointestinal infections

As already highlighted, oral bacteria ( *e.g. Corynebacterium pseudodiphtheriticum*, *Fusobacterium nucleatum*, *Nocardia* spp., *Prevotella melaninogenica*, *S. aureus*, *S. epidermidis*, *Veillonella* spp.) are vital for the reduction of salivary nitrate to nitrite as part of the entero-salivary circulation; nitrite is further reduced to NO. ^113^ This represents a symbiotic relationship between bacteria and the mammalian host; the host provides the nutrients and the environment in return for nitrite production, ^76^ as in the absence of oxygen, nitrate and nitrite are commonly used by such bacteria as terminal electron acceptors for respiration. ^75^

Oral consumption of nitrate and the resulting increase in nitrite in the oro-pharynx leads to salivary alkalinisation (pH ~7.0 to 7.5) ^114^ and so reduction in detrimental bacteria and caries. ^74^ Similarly, nitrate supplementation was associated with increased oral *Rothia* spp. and *Neisseria* spp, and diminished oral *Prevotella* spp. and *Veillonella* spp.; in parallel, plasma nitrite levels rose and systemic blood pressure fell. ^115^ Salivary nitrite production is related to the abundance of oral-nitrate-reducing bacteria. ^116^ In contrast, bacteria and yeast, in particular *Lactobacillus* spp., *Streptococcus* spp. and *C. albicans*, are key to the development of dental caries through the production of acid. Equally, antibiotics that kill nitrate-reductase-containing bacteria inhibit oral nitrite production and so increase the risk of oral thrush. ^117^ Acidified nitrite has antibacterial activity against *Helicobacter pylori in vitro*, ^118^ an experiment that likely mimics the scenario seen by these bacteria in the stomach after a nitrate/nitrite-rich meal.

### Cutaneous infections

The skin is a potent source of nitric oxide and production is increased with exposure to sunlight (specifically ultraviolet radiation) sufficient to lower blood pressure. ^119^^,^^120^ Hence, skin-derived NO may form a natural dermatological antimicrobial defence. Numerous studies have demonstrated that topical acidified sodium nitrite reduces cutaneous infections due to a variety of viruses and bacteria ( Table 7) although prophylaxis had to be continued in some cases since NO suppressed replication without necessarily being viro-toxic. ^121^^–^^126^ Inorganic nitrate has been used for the treatment of human papilloma virus. ^127^ Phase II clinical trials have found that acidified nitrite in cream reduced *Leishmania major/tropica* amastigotes and promastigotes with a reduction in cutaneous leishmaniasis ^123^ and increased cure rates in tinea pedis. ^121^ Novel NO agents are in development to treat skin conditions ( Table 8).

**Table 8.** Examples of commercial development of novel nitric oxide donors/agents with efficacy against target disease and microbes (where relevant, last searched 15 March 2021).

	Target organism/disease	Commercial company
NO gas for inhalation		Beyond Air
NO gas for inhalation		INOmax
NO-releasing solution for nebulisation (sodium nitrite and citric acid)	COVID-19	30 Technology
NO released from acidified nitrite via a semi-permeable membrane	Cutaneous *S. aureus, E. coli* ^249^	
NO macromolecular scaffolds, ^46^^,^^250^ e.g. NO-releasing cyclodextrins ^251^	*P. aeruginosa*	Vast Therapeutics
Polymer-based chronic NO delivery systems e.g. for treatment of biofilms, ^252^	Cutaneous viruses (human papilloma virus, molluscipoxvirus), dermatophytes ( *Epidermophyton floccosum*, *Fusarium solani*, *T. rubrum, T. menatgrophytes*) or yeast ( *Candida albicans/tropicalis*, *Malassezia furfur*) ^239^	Novan
NO-releasing solution/gel ( NORS2791) NO-releasing solution ( NORS6491) NO-releasing nasal spray (NORS1002) NO-releasing nasal lavage (NORS4002)	Acne ( *Propionibacterium*), fungal nail infections/onychomycosis ( *T. rubrum*, *T. interdigitale*) Athlete’s foot ( *T. rubrum* and *T. mentagrophyte*) Cold, ‘flu, COVID-19 Sinusitis	SaNOtize
NO-stimulating nasal spray (GLS-1200)		GeneOne Life Science
Nitric oxide generating lozenges (sodium nitrite)	COVID-19	Nitric Oxide innovations
NO-generating probiotic patches, e.g. based on Lactobacilli conversion of glucose to lactic acid, and acidification of sodium nitrite ^221^^,^^245^	*S. aureus*	McGill University, Canada
Nitroreductase-activated release of NO, e.g. by O ^2^-(4-Nitrobenzyl) diazeniumdiolate ^253^ or nitroaromatic-protected piperazine diazeniumdiolate ^254^	*E. coli*	Indian Institute of Science Education and research, Pune, India Colorado State University, Fort Collins, USA

### Respiratory infections

In animal and human experiments, NO substrate (L-arginine) and a NO donor (SNP) has been shown to improve the mucociliary activity of the upper respiratory tract ^128^ suggesting a modulatory role for NO in nasal barrier function and clearance. Novel NO agents building on this observation are in development ( Table 8):

Endogenous NO has potent pulmonary haemodynamic and bronchodilator effects physiologically. The importance of endogenous NO in preventing infection is apparent experimentally where inhibition of NO results in increased susceptibility to microbes including *Leishmania* spp., *Mycobacterium* spp. and *Plasmodium* spp. ^129^ Similarly, NO sources are used therapeutically, for example sildenafil in the management of pulmonary hypertension ( Table 7). In respect of airway epithelial cells, nitrite reduced *P. aeruginosa* biofilm growth. ^130^ In infection, NO reduces pulmonary vascular resistance and intrapulmonary shunt, and improves oxygen partial pressure in patients with acute severe pneumonia. ^131^ More specifically, inhaled NO improves arterial oxygenation enabling a reduction in inspired oxygen therapy and airway pressure support, and reduces lung infiltrates, in patients with severe acute respiratory syndrome (SARS). ^31^ These findings continue after termination of NO therapy suggesting that NO has both pulmonary vasodilator and anti-SARS effects. Small uncontrolled clinical studies have suggested that iNO may be beneficial in COVID-19. ^132^^–^^136^ iNO and novel NO agents are in development, primarily for COVID-19 at present ( Table 8, 9).

**Table 9.** Ongoing or planned trials of NO sources for prophylaxis or treatment of COVID-19 (also see ^255^).

Trial name (registration)	NO source	Location	Design	Phase in population	Outcome	Sites N	Rx days	Finish
**C19 prevention**								
BEET-Winter (ISRCTN51124684)	Nitrate juice, oral	UK	Cluster DBPC	Phase II in care homes	New infection (any, including C-19)	30	60	06/21
Berra *et al.* (NCT04312243)	NO gas, inhaled	USA	CCS	Phase II in healthcare staff	New C-19 diagnosis	1 470	14	03/21
(NCT04408183)	GLS-1200 nasal spray (NO stimulant)	USA	DBPC	Phase II	Adverse events PCR-positive infection	2 225	28	03/21
**C19 treatment**								
Fiorentino *et al.* (NCT04637906)	L-arginine, oral	Italy	RCT DBPC	Phase II in hospital: on oxygen	Normalisation of P/F fraction	1 290	60	09/21
(NCT04570384)	L-citrulline, iv	USA	DBPC	Phase II in hospital: on oxygen	[L-arginine]	1 60	10	12/21
CACOLAC (NCT04404426)	L-citrulline, iv	France	DBPC	Phase II in ICU and ventilated for ARDS	SOFA score	1 32	7	09/21
COVINOX (NCT04421508)	NO gas, inhaled	USA	DBPC	Phase III in hospital: on oxygen	Death or respiratory failure	? 500	?	06/21
NOCOVID (NCT04305457)	NO gas, inhaled	China Italy USA	RCT sham	Phase II in hospital: Moderate C-19	Mechanical ventilation	3 70	28	03/21
NoCovid (NCT04290858)	NO gas, inhaled	China	RCT, open	Phase II in hospital: Moderate C-19	Need for intubation-ventilation	1 400	14	02/22
NOSARSCOVID (NCT04290871)	NO gas, inhaled	China Italy USA	RCT sham	Phase II in hospital: severe C-19, PaO _2_/FiO _2_ <300 on air	PaO _2_/FiO _2_ > 300 on air	4 200	14	03/21
Somberg *et al*. (NCT04601077)	NO lozenge, oral	USA	RCT DBPC	Phase II in early COVID-19 in African-Americans	Hypotension, hospitalisation	1 100	30	07/21
COVID-IND-02 (NCT04443868)	NO nasal spray/irrigation	USA	RCT DBPC	Phase II in mild COVID-19	Duration of infectivity	? 50	14	07/21
NOCOVID (NCT04337918)	NO nasal spray/irrigation	Canada	RCT open	Phase II in workers at high risk of exposure	New C-19 diagnosis	5 143	14	09/20
(NCT04460183)	Nebulised sodium nitrite and citric acid (RESP301)	UK	RCT, open	Phase II/III hospitalised COVID-19	Progression in WHO scale by ≥ 1 point	4 300	14	04/21
Sildenafil (NCT04304313)	Sildenafil, oral	China	Open	Phase II in hospital: pneumonia	Disease remission	1 10	14	11/20

ARDS: adult respiratory distress syndrome; C-19: COVID-19; CCS: case-controlled study; DBPC: double-blind placebo-controlled; iv: intravenous; RCT: randomised controlled trial; SOFA: sequential organ failure assessment; TBC: to be confirmed.

Dipyridamole, a phosphodiesterase 5 inhibitor, may also have similar beneficial effects in severe COVID-19. ^33^ A phase II clinical trial found that L-arginine might have beneficial effects when given on top of conventional therapy for tuberculosis ( Table 7). ^48^

### Urinary tract infections

There may also be a role for dietary nitrate/inorganic nitrite in the prevention and treatment of urinary tract infections. Many of the lower urinary tract opportunistic organisms ( *e.g. E. coli*) possess nitrate reductases, this forming the basis of urine dipstick detection of nitrite. In acidic urine conditions, nitrite is reduced to NO with toxicity to bacteria; for example, transferring nitrite-rich urine containing *E. coli* to a more acidic environment ( *e.g.* pH 5.5) dose-dependently inhibited bacterial growth, ^137^ an effect potentiated by vitamin C. The antibacterial potency is comparable to conventional antibiotics such as trimethoprim and nitrofurantoin.

This approach has been tested by filling urinary catheter retention balloons with nitrite and ascorbic acid, resulting in measurable amounts of NO outside the membrane and effectively killing two strains of *E. coli* in the surrounding urine. ^138^ A similar approach found decreased bacterial counts and prevented biofilm formation by *P. aeruginosa*, *K. pneumoniae*, and *Enterobacter cloace* (but not *E. coli* or *S. aureus*). ^139^

Last, instillation of bacillus Calmette-Guerin (BCG, an attenuated strain of *Mycobacterium bovis*) into the bladder is used for the treatment of superficial/non-muscle invasive bladder cancer and carcinoma *in situ.* BCG induces long-term increases in NOS activity in urothelial cells ^140^^,^^141^ and the formed NO is toxic to the malignant cells. The use of BCG to provide non-specific protection against SARS-CoV-2 is to be tested ^142^^,^^143^ although vaccination in infancy does not appear to protect against COVID-19 in adults. ^144^

### Other infections

Nitrate (usually KNO _3_) and nitrite (NaNO _2_) have been used for millennia to preserve food, especially meat and fish. ^145^ Food preparation leads to reduction of nitrate to nitrite, and nitrite inhibits bacterial growth, especially *Clostridium botulinum*, a key and severe cause of neurotoxin poisoning. Additionally, nitrite adds colour to food, ^58^^,^^146^ flavour (in part by overcoming rancid tastes) and is an antioxidant.

NO donors have also been investigated for eradicating or dispersing biofilm organisms. For example, GTN synergises with citrate and ethanol in eradicating biofilms (related to *S. aureus*, MRSE, *P. aeruginosa* and *C. albicans*) in an experimental catheter lock model. ^147^ Similarly, isosorbide mononitrate synergised with antibiotics to disperse then kill *S. aureus.* ^148^ An NO-releasing contact lens has been developed to treat microbial keratitis due to *P. aeruginosa* and *S. aureus*). ^149^

GTN may have improved outcome after infection in participants enrolled into the RIGHT-2 trial, a study where paramedics recruited patients with suspected stroke and randomised them to GTN versus sham. Overall, the trial was neutral. ^150^ However, in a planned subgroup analysis of those participants with a final diagnosis of a non-stroke mimic, functional outcome was better with GTN. ^150^ In a *post hoc* analysis of participants in this subgroup, GTN was associated with a better outcome in those with a final diagnosis of infections of the respiratory and urinary tracts which raises the possibility that NO was treating the infectious cause underlying the stroke mimic diagnosis.

### The prostaglandin-cyclic adenosine-phosphodiesterase-3 system

As with the NO system, the prostaglandin-cyclic adenosine monophosphate-phosphodiesterase-3 (PG-cAMP-PDE3) system has similar vasculo-protective roles with anti-leucocyte, antiplatelet and anti-smooth muscle, and pro-endothelial effects. It is therefore interesting to note that prostaglandins (PGA _1_, PGJ _2_), including prostacyclin (PGI2 and analogues), have been reported to have antiviral effects. ^151^^–^^154^ Whether drugs based on these ^155^ or the PDE3 inhibitor, cilostazol, have efficacy against SARS-CoV-2 remains to be investigated. Further, endogenous NO and PGI _2_ work together in the vascular tree, and it is conceivable that their potential antimicrobial effects will similarly synergise. Their combination, in the forms of ISMN and cilostazol, have been tested after stroke ^156^ but not yet reported for the prevention or treatment of infection.

### Interaction between nitric oxide and vaccine efficacy

The interaction between diet, nutrition state and vaccine effectiveness has been assessed in multiple studies, principally in low–middle income countries where vaccination is paramount, especially in children, and yet where malnutrition may be widespread. In a systematic review and meta-analysis of observational studies and randomised controlled trials, there was little suggestion that malnutrition had any effect on vaccine responses ^157^; similarly, supplementation with vitamins and D, and iron and zinc, did not appear to modify responses. In preclinical studies, protein-energy malnutrition had limited influence on vaccine efficacy in mice. ^158^ The effect of dietary nitrate levels on vaccine efficacy is unstudied.

If nitric oxide derivatives attenuate microbial infections, then the efficacy of vaccines based on live attenuated viruses and bacteria (such as measles, poliovirus, BCG) might be attenuated by treatment with NO. Although there are many factors known to alter vaccine effectiveness ( *e.g.* age), the effect of NO has not been studied.

### Post-infection morbidity

Many infections cause long-term morbidity with chronic fatigue syndrome (CFS) and symptoms including fatigue, tiredness, myalgia, cognitive impairment and depression. Example associated microbes include *Borrelia burgdorferi* (Lyme disease), *Chlamydia pneumoniae* (community acquired pneumonia), Epstein–Barr virus (infectious mononucleosis), human herpes virus 6 (exanthema subitem), human immunodeficiency virus (AIDS), polio virus, SARS-CoV-1 virus (SARS), SARS-CoV-2 (long-COVID) and West Nile virus (fever). ^159^^–^^161^ Although CFS may represent chronic or latent infection, it is more likely to reflect the presence of post-infectious chronic inflammation. Hypothetically, these patients might benefit from inorganic nitrates in view of their positive effects on exercise performance (elite athletes take beetroot juice for this purpose) and cognition, ^73^^,^^92^ and potentially antimicrobial effects, a question that needs addressing ( Table 7). A phase II trial of L-citrulline is studying this approach in patients with post-polio syndrome. ^162^

### Excess nitric oxide during infection

During severe infection, sepsis (defined as “life-threatening organ dysfunction caused by a dysregulated host response to infection”) often develops. Septic shock is a subset of sepsis and is a leading cause of death worldwide. ^163^ It manifests as hyper- or hypo-pyrexia, altered mental state, hypotension, tachycardia, tachypnoea, hypoxia, anuria and/or lactataemia. This can occur with many infections due to:

• Gram negative bacteria: *Bacteroides fragilis*, *C. pneumoniae*, *Enterobacter* spp., *E. coli, Haemophilus influenzae*, *Klebsiella* spp., *Legionella* spp., *Neisseria meningitidis*, *Proteus* spp., *P. aeruginosa*, *Yersinia pestis.* ^61^
• Gram positive bacteria: *Clostridium* spp., *Enterococcus* spp., *Listeria monocytogenes*, *Staphylococcus* spp., *Streptococcus agalactiae/pneumoniae/pyogenes*
• Viral: Adenovirus, Coronaviruses, Dengue viruses, Ebola virus, Enteroviruses, human immunodeficiency virus, Influenza virus (A and B), haemorrhagic fever viruses, Parechoviruses. ^164^^,^^165^
• Fungi: *Candida* spp. ^166^
• Protozoa: *Plasmodium falciparum*, *Schistosoma mansoni.* ^167^

**Figure 1.**
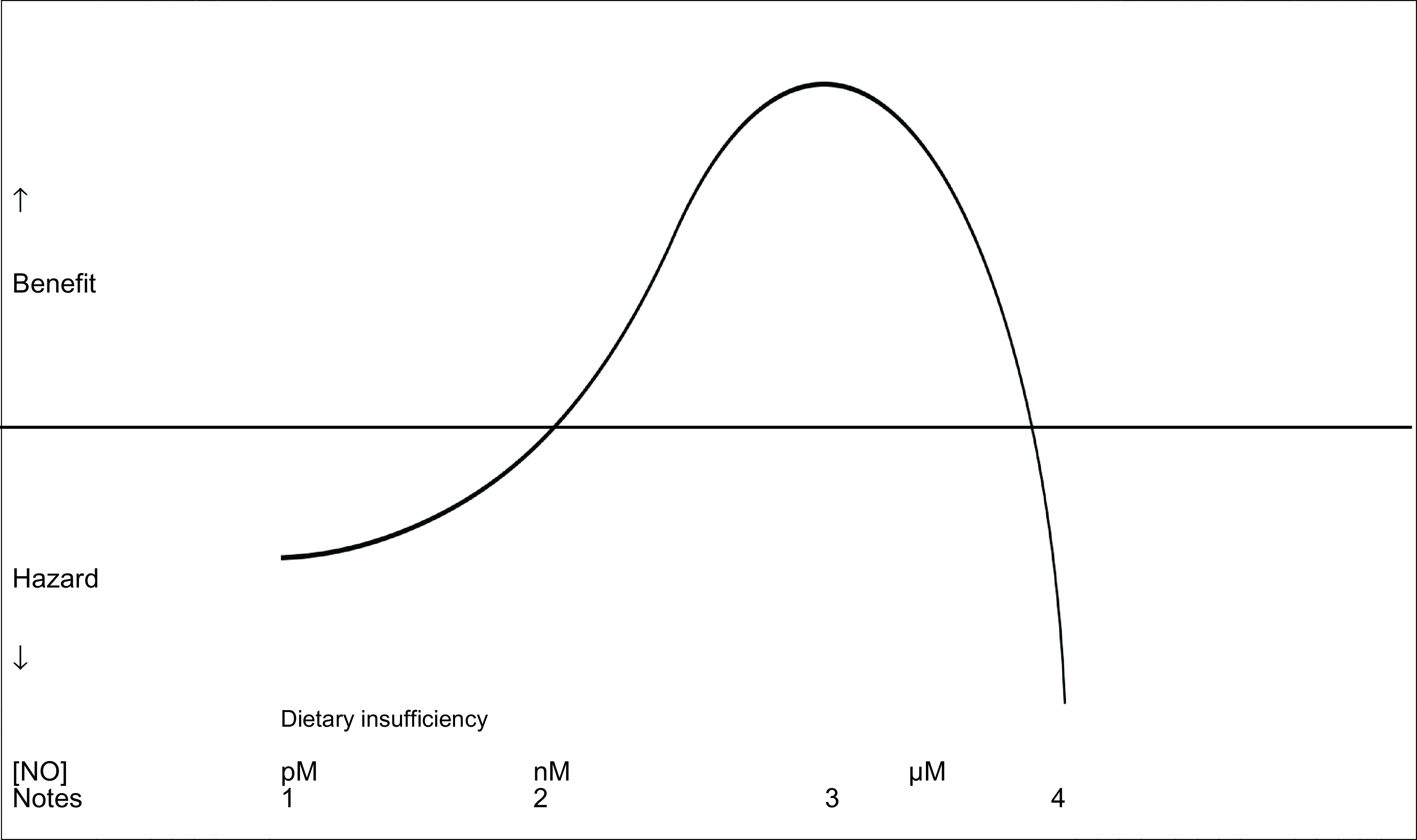
Schematic of concentration response curve for antimicrobial effects of nitric oxide. 1.Reduced eNOS-derived NO related to dietary insufficiency, older age, vascular disease2.Normal eNOS-derived vascular NO3.iNOS-derived NO or low/moderate dose exogenous NO source4.iNOS-derived NO in septic shock or high dose exogenous NO source

Typically, autoamplification of circulating cytokines (so-called cytokine storm) leads to excess NO synthesis, mostly derived from inducible NOS, leading to high circulating NO levels and the development of septic shock. In these circumstances, treatment with exogenous NO might be inappropriate. Trials of inhibiting endogenous NO synthesis with NOS-inhibitors in critically ill patients with sepsis have been reported although, disappointingly, did not improve outcome; indeed, the non-specific NOS-inhibitor, NG-methyl-L-arginine hydrochloride (L-NMMA, 546C88), was associated with increased death. ^168^ It is not clear why inhibiting NO synthesis was ineffective but non-selective NOS inhibitors were used meaning that both toxic (iNOS) and beneficial (eNOS) sources of NO were inhibited; pharmacologically, such inhibitors will have reduced cardiac output, organ perfusion and tissue oxygenation. In the absence of licensed selective iNOS inhibitors, perhaps the analogous approach used in the management of hyperthyroidism using block (with carbimazole) and replace (thyroxine) might be effective, *i.e.* block NOS activity and replace with a low dose of a NO donor. That excess NO is dangerous does not mean that pharmacological doses of NO cannot be effective ( Figure 1, Tables 6, 11) since all effective interventions in medicine have an inverted “U” dose response.

Some infections have opposing *in vitro* and *in vivo* responses to NO. For example activated macrophage-derived NO or NO donors such as SNAP reduced *Trypanosoma brucei* proliferation *in vitro* ^169^^,^^170^ whereas endogenous iNOS-derived NO suppressed protozoa-antigen specific T-cell proliferative responses and so worsened infection, at least in infected mice. ^170^ Intracellular protozoal infections are unlikely to be affected in this manner since macrophage-derived NO would be able to act directly on pathogens such as *Leishmania major.* ^171^

Other infections do not appear, at least *in vitro*, to induce iNOS. For example, *Cryptococcus neoformans* failed to induce NOS in primed macrophages, ^172^ apparently due to a lack of TNF-α secretion, probably because the polysaccharide capsule masked the signal for TNF-α secretion. Interestingly, non-encapsulated mutants of *C. neoformans* did induce endogenous NOS.

High levels of iNOS activation were antimicrobial in studies of malaria. Based on monocyte-derived mRNA levels in circulating blood, uncomplicated malaria was associated with increased levels of iNOS activation in contrast to patients with severe malaria who had lower levels. ^173^

### Epidemics and pandemics

Over recorded history, most epidemics and pandemics have resulted from viral infections including Ebola (viral haemorrhagic fever), influenza (H1N1, H2N2, H3N2, H3N8), HIV-1 (AIDS), polio (poliomyelitis), smallpox, yellow fever, zika or corona (OC43, MERS-CoV, SARS-CoV-1/2) viruses. Bacterial pandemics have resulted from *Vibrio cholerae* (cholera), *S. enterica* (typhoid fever) and *Yersinia pestis* (plague). Studies *in vitro* have reported findings suggesting that NO can reduce infection for some of these pathogens ( Tables 2, 3) but information appears to be lacking for smallpox, yellow fever, zika and cholera ( Table 10).

**Table 10.** Future research questions relating to nitric oxide therapy for microbes.

Question	Microbe	*In vitro*	*In vivo*	Clinical trials
What effect does NO have on so-far unstudied pandemic microbes?	Viruses: smallpox, yellow fever, Zika. Bacteria: *Vibrio cholerae*	+		
What effect does NO have on so-far unstudied other microbes?	Bacteria: Mycoplasma. Archaea.	+	+	
What effect does NO have on micro/macro-thrombosis?	Viruses: Ebola. Influenza. MERS. SARS-CoV-1/2		+	+
What is the effect of timing on outcome in prevention or treatment of mild and severe disease?	Any			
Could NO be used as a non-specific adjuvant to antimicrobial therapy (where septic shock is absent)?	Any		+	+
Is the strategy of block (NOS inhibitor) and replace (NO donor) effective in septic shock?	Intensive Care Unit infections		+	+
Do NO sources, e.g. dietary nitrate or NO donors, prevent/treat/improve outcome after COVID-19?	SARS-CoV-2			+
Do NO sources, e.g. dietary nitrate or NO donors, prevent and treat outcome after endemic ‘flu?	SARS-CoV-2			+
Do combined NO and PG sources have agonistic antimicrobial effects?	Any	+	+	+
Do NO sources reduce the efficacy of live attenuated vaccines?	Measles virus, poliovirus, bacilli Calmette-Guerin		+	+
Do NO sources reduce chronic symptoms and improve quality of life after infection?	Lyme disease			+

NO: nitric oxide; PG: prostaglandin.

**Table 11.** Balance between potential beneficial and hazardous effects of NO sources in preventing and treating infections.

	Benefit	Ineffective/Hazard
*In vitro*	Considerable static/cidal data	Limited neutral/negative data suggesting that there may be publication bias
*In vivo*	Some static/cidal data	
Clinical	Some positive phase II trials	
Concentration	Moderate	Low or very high (as in septic shock although NOS inhibitors ineffective)
		Microbial resistance to NO, *e.g.* synthesis of NO to resist oxidative stress
		Organic nitrates generate reactive NO species

With multiple pandemics over the last 100 years, it is only inevitable that further ones will occur and some, like COVID-19, will comprise a “global catastrophic biological risk”. ^174^ Global pandemics will most likely be caused by a respiratory-spread virus that crosses over from animals such that humans have no inherent immunity to it. Likely candidates include orthomyxoviruses (especially influenza A viruses such as H7N9), paramyxoviruses ( *e.g.* measles, mumps, croup), pneumovirus ( *e.g.* human metapneumovirus), coronaviruses and picornaviruses (especially rhinoviruses and enteroviruses). All of these have had strains that have crossed from animals to humans. This emphasis on RNA viruses is because DNA viruses tend to have lower mutation rates and, therefore, evolve more slowly and are less likely to escape the human immune system within the first rounds of infection. Nevertheless, DNA viruses, such as pox or herpes viruses from great apes or monkeys, do have the potential to jump species. Non-viral causes of pandemics are less likely since most bacteria will be treatable with broad-spectrum antibacterial agents, most fungi are thermally restricted, and prions would require massive food contamination (and only spread slowly). ^174^ Protozoa are usually thermally restricted although global warming may allow malaria to spread more widely in temperate zones.

Unfortunately, pandemics/epidemics may co-exist as seen with SARS-CoV-2 and dengue in Brazil, ^175^ and both with *S. enterica* in Pakistan; ^176^^,^^177^ in part, this reflects increasing travel with aircraft providing a portal for numerous microbes. ^178^ Of theoretical concern was the potential for COVID-19 and epidemic influenza to co-exist during winter in the Northern hemisphere, this possibly leading to a dramatic increase in deaths. ^179^ Nevertheless, ‘flu rates were very low in both southern and northern hemisphere 2020 winters, presumably due to hands, face, space, mask and fresh air measures. All-in-all, the absence of a true broad-spectrum of antiviral agents is a major concern ^180^ and a potential agent such as NO with antimicrobial effects that extend beyond viruses would be most welcome.

### Implications for SARS-CoV-2 and COVID-19

One possible explanation for the observation that COVID-19 outcomes are worse in older people, males, black or Asian ethnicity, and those with co-morbidities such as diabetes, hypertension, stroke and chronic lung disease, ^181^ is that these groups have lower vascular NO activity ^4^^,^^5^^,^^9^ and so mount a sub-optimal host response against infection. Increasing NO availability is therefore a potential therapeutic strategy. Several NO sources have potential relevance to preventing and treating COVID-19. L-arginine, sodium nitrite, GTN, SNP, NO and dipyridamole each have clinical antimicrobial activity and can be administered, variously, orally, intravenously or as NO gas in the intensive care unit. Transdermal GTN, and oral ISMN, dipyridamole and sildenafil may be administered in the community or hospital. Of these, NO gas, dipyridamole and sildenafil are already being tested for preventing or treating COVID-19 ( Table 9). It remains to be determined if increasing dietary nitrate may be a cost effective and safe intervention of widespread health relevance for the prevention of COVID-19 and, indeed, other emerging, pandemic, epidemic or endemic infections. Recent trial evidence provides indirect supporting evidence for the potential anti-SARS-CoV-2 effect of NO. First, dexamethasone and tocilizumab reduced death in patients in intensive care units, ^182^^,^^183^ and these agents and NO share anti-inflammatory effects. And second, interferon-ß reduced the need for intensive care in COVID-19 patients; ^184^ type I interferons increase iNOS activity and so have antimicrobial effects, as seen with *L. major* and *Burkholderia pseudomallei.* ^185^^,^^186^

## Discussion and conclusions

Nitric oxide is a fundamental molecule with wide-ranging and potent vascular, anti-platelet, anti-inflammatory and tumoricidal effects. Further, there is a large volume of literature spanning the last 30+ years demonstrating that NO also has potent *in vitro* antimicrobial effects on a wide variety of viruses, bacteria, protozoa, fungi and yeasts; these are supported by a modest number of *in vivo* studies. Further, several positive clinical phase II trials of NO have been reported in viral, bacterial, protozoa and fungal infections, these relating particularly to skin and respiratory infections administered by cream and gas respectively. Although not from randomised trials, there is also evidence that dietary nitrate modifies the oral biome and so reduces dental caries.

However, these results cannot be considered persuasive on their own. First, few neutral or negative studies have been reported suggesting that there may be a risk of publication bias. Second, conflicting data in some dual-protocol studies with positive *in vitro* and neutral/negative *in vivo* data suggest that although NO is antimicrobial *per se*, the local tissue environment may overcome or reverse this effect. Third, organic nitrates can suffer from tolerance and may lead to the generation of reactive NO species such as peroxynitrite and S-nitrosothiols which might exacerbate rather than attenuate infection. Fourth, resistance may develop although this seems unlikely to be a generic issue, not least because NO levels can change, and be changed, much faster than any microbe can raise defensive mechanisms. Fifth, some microbes can produce their own NO and use this to resist the oxidative stress induced by external NO and its derivatives. Sixth, excess NO production is associated with the development of septic shock which might suggest that any NO is ineffective. Potentially, unsuccessful trials of non-selective NOS-inhibitors in severe sepsis may have confused the issue, perhaps by suggesting that treatment with NO is not important in infection. Seventh, positive clinical studies have been performed in environments where very high local concentrations of NO can be achieved and without the risk of reactive responses, in particular on the surface of tissues such as cream on the skin, dietary nitrate in the mouth, nitrite in the stomach, NO gas in the lungs and nitrite in the bladder; whether NO is effective as an antimicrobial within tissues and the vascular tree remains to be determined.

There are many sources of NO suitable for studying the prevention and treatment of milder infections in the community and hospital ( *e.g.* topical sodium nitrite, oral NO donors such as ISMN, or oral PDE5 inhibitors such as dipyridamole or sildenafil), and treatment of serious infections in hospital ( *e.g.* intravenous L-arginine, sodium nitrite or NO donors such as GTN or SNP, of NO gas). NO may also be delivered *via* a high nitrate diet, thus offering a widely available and inexpensive public health approach to potentially reducing and attenuating the severity of infections worldwide. This approach has the added advantage that such diets are already known to reduce vascular disease and some cancers, and possibly other inflammatory diseases and dementia.

In summary, the wealth of *in vitro* data suggest that NO has generic antimicrobial effects. However, some data suggest that NO may be ineffective or even hazardous and these reinforce our view for the need for large scale clinical trials of NO donors in the community and hospitals to prevent and treat infections. Although such studies need to focus urgently on the COVID-19 pandemic (especially with the lack of broad spectrum antiviral agents ^180^), other pathogens also need to be targeted. However, patients with established septic shock should not be administered NO donors to avoid exacerbating vascular collapse. One utopian vision would be demonstration that high dietary nitrate intake produces pre- or post-exposure prophylaxis against infections and their severity in the community whilst NO donors are effective antimicrobial treatments for use by general practitioners and in hospitals.

## Data availability

No data are associated with this article.
