# Supplementary material for: Nitric oxide for the prevention and treatment of viral, bacterial, protozoal and fungal infections
Source: F1000Res. 2021 Oct 22;10:536. Originally published 2021 Jul 5. [Version 2] doi: 10.12688/f1000research.51270.2 (PMC9171293; doi:10.12688/f1000research.51270.2)
Supplement: Peer review history

# Peer review: Reviewer response for version 2

**DOI:** 10.5256/f1000research.78470.r138632

**Published:** 2022-06-06

**Authors:** Steffen B. Wiegand

**Affiliations:** 1 Anesthesia Center for Critical Care Research of the Department of Anesthesia, Critical Care and Pain Medicine, Massachusetts General Hospital, Harvard Medical School, Boston, MA, USA; 2 Department of Anesthesiology, Critical Care and Pain Medicine, University Hospital, LMU, Munich, Germany

Nitric oxide (NO) has been used in many different clinical settings over the years. The approval of inhaled NO for the treatment of pulmonary hypertension of the newborn has been a game changer and has since then saved many lives. NO has failed to prove a significant outcome improvement in patients suffering from Acute Respiratory Distress Syndrom (ARDS).

 In this review, the authors have focused on the not-widely known broad antimicrobial role of NO. Although the antimicrobial effects are known for more than two decades, there has not been a systematic review of the topic. In addition to the well-written and compelling text, I have some suggestions, which I believe will add to the review.

 Inhalation of NO is a main source of exogenous NO and has been used for almost three decades. For a long time, the administration of inhaled NO has been limited to mechanically ventilated patients in the ICU. However, in recent years the development of devices enabling pulsed generation of NO out of ambient air and devices developed during the surge of SARS-CoV2 allow the use in awake patients. A recently published review summarizes the different delivery methods of inhaled nitric oxide in intubated and non-intubated patients and should be added to this review ^1^.

 Another important aspect worth mentioning in a separate paragraph is the use of inhaled nitric oxide in patients suffering from cystic fibrosis. These are a special patient group for studying new therapeutic approaches. The lungs of these patients are often colonized by multiresistant hard-to-treat gram negative bacteria such as burkholderia and pseudomonas species. ^2^^,^^3^^,^^4^

 In this context, it is worth mentioning that there seems to be some evidence that inhaled NO, in addition to its beneficial effect on mucociliary clearance, dispersal of biofilm, and bronchiolysis, improves antibiotic susceptibility in resistant bacteria. ^5^^,^^6^

 One limitation of high dose NO treatment is the production of methemoglobin. However, there are different strategies to minimize methemoglobin levels. Intermittent delivery of inhaled NO helped to minimize metHb concentrations. In addition, in-vitro data suggest that intermittent delivery of high dose NO increases the overall antibacterial effect. ^7^

 Another approach is the co-administration of high dose NO and methylene blue, which reduces methemoglobin to hemoglobin and helps limit methemoglobin production, and allowed longer high dose NO administration. ^8^

 A very compelling clinical use might be the administration of inhaled NO as a prevention therapy for infections of the lung in explanted lungs in the context of a lung transplant. Ex-vivo perfusion and co-administration of high dose inhaled NO, similar to a high dose of Colistin e.g. during transportation might be a novel strategy to reduce complications after transplantation. ^9^

Is the review written in accessible language?

Yes

Are all factual statements correct and adequately supported by citations?

Yes

Are the conclusions drawn appropriate in the context of the current research literature?

Yes

Is the topic of the review discussed comprehensively in the context of the current literature?

Yes

Reviewer Expertise:

ARDS, Immunology

I confirm that I have read this submission and believe that I have an appropriate level of expertise to confirm that it is of an acceptable scientific standard, however I have significant reservations, as outlined above.

## References

[1] : Inhaled Nitric Oxide Delivery Systems for Mechanically Ventilated and Nonintubated Patients: A Review. *Respir Care* .2021;66(6) : 10.4187/respcare.08856 1021-1028 10.4187/respcare.08856 34039761PMC10408459

[2] : Gaseous nitric oxide to treat antibiotic resistant bacterial and fungal lung infections in patients with cystic fibrosis: a phase I clinical study. *Infection* .2016;44(4) : 10.1007/s15010-016-0879-x 513-20 10.1007/s15010-016-0879-x 26861246

[3] : Compassionate Nitric Oxide Adjuvant Treatment of Persistent Mycobacterium Infection in Cystic Fibrosis Patients. *Pediatr Infect Dis J* .37(4) : 10.1097/INF.0000000000001780 336-338 10.1097/INF.0000000000001780 28885458

[4] : High-Dose Inhaled Nitric Oxide as Adjunct Therapy in Cystic Fibrosis TargetingBurkholderia multivorans. *Case Reports in Pediatrics* .2020;2020: 10.1155/2020/1536714 1-6 10.1155/2020/1536714 PMC733476532685229

[5] : Exogenous Nitric Oxide Improves Antibiotic Susceptibility in Resistant Bacteria. *ACS Infectious Diseases* .2021;7(1) : 10.1021/acsinfecdis.0c00337 23-33 10.1021/acsinfecdis.0c00337 33291868

[6] : Morphological analysis of the antimicrobial action of nitric oxide on gram-negative pathogens using atomic force microscopy. *Acta Biomater* .2009;5(5) : 10.1016/j.actbio.2009.01.025 1405-15 10.1016/j.actbio.2009.01.025 19250890PMC3574578

[7] : Antimicrobial effects of nitric oxide in murine models of Klebsiella pneumonia. *Redox Biol* .39: 10.1016/j.redox.2020.101826 101826 10.1016/j.redox.2020.101826 33352464PMC7729265

[8] : A novel pre-clinical strategy to deliver antimicrobial doses of inhaled nitric oxide. *PLoS One* .2021;16(10) : 10.1371/journal.pone.0258368 e0258368 10.1371/journal.pone.0258368 34644318PMC8513841

[9] : Safety of continuous 12-hour delivery of antimicrobial doses of inhaled nitric oxide during ex vivo lung perfusion. *J Thorac Cardiovasc Surg* .2022;163(3) : 10.1016/j.jtcvs.2020.11.150 841-849.e1 10.1016/j.jtcvs.2020.11.150 33478833

# Peer review: Reviewer response for version 2

**DOI:** 10.5256/f1000research.78470.r138631

**Published:** 2022-05-30

**Authors:** Jan Mohammad Mir

**Affiliations:** 1 Department of Chemistry, Islamic University of Science and Technology, Awantipora, India

The manuscript under review represents a study of potential interest. Though several review articles of similar themes have already been published. This article however introduces NO as a one-man army for broad-spectrum bioactivity.  Therefore, I suggest the following modifications to make the article more interesting to the readership:

1. Introducing lines in the introduction must be about human physiological effects of NO, and later defense by microbes and defense against microbes using NO be highlighted.
2. NO is a pleiotropic signaling molecule: given in the introduction should be made more comprehensive, and distinctive by using "Gasotransmitter" concept as well (reference ^1^). 
3.  2 ^nd^ paragraph “As people age, endothelial-derived vascular NO levels fall and so vascular function declines causing relative endothelial dysfunction, pro-platelet, and pro-inflammatory effects, and increased smooth muscle proliferation.” needs literature support.
4. NO is a small diatomic hydrophobic colourless gas that diffuses easily and has a short half-life. Specify this line...
5.  "In reality, the chemistry of NO is more complex and it exists in several redox forms: nitrosonium cation (NO ^+^), NO (NO ^•^), and nitroxyl anion NO ^−…^………………." Discuss metabolic or biological fate only.
6. This part is suitable for introduction “ *As an inorganic molecule, its central role in biology as a signalling molecule was only discovered in the 1980s, ^24^ the identification of which led to the Nobel Prize for Physiology and Medicine in 1998. ^25^ Ironically, the medicinal use of NO in the form of glyceryl trinitrate (GTN) for angina prophylaxis antedates the modern understanding of the biological synthesis and role of NO by more than a century. ^26^*”
7. The chemistry and biology section needs updating. 
8. The most important/major modifications: give the colourful schemes showing the mechanism of action of NO against the target diseases. Also, the introduction, main discussion, and conclusion must be redrawn to highlight "NO as a treatment option", the clinical trials discussion, the use of several NORMS (NORM-1, NORM-2, etc), the effect of NO-carriage system, Natural NO-sources, synthetic NO-sources with limitations, Inorganic versus organic forms, etc (eg., in the review publications as given ^2^^,^^3^.

Is the review written in accessible language?

Yes

Are all factual statements correct and adequately supported by citations?

Partly

Are the conclusions drawn appropriate in the context of the current research literature?

Partly

Is the topic of the review discussed comprehensively in the context of the current literature?

No

Reviewer Expertise:

Coordination, Bioinorganic  and Computational Chemistry.

I confirm that I have read this submission and believe that I have an appropriate level of expertise to confirm that it is of an acceptable scientific standard, however I have significant reservations, as outlined above.

## References

[1] : A gentle introduction to gasotransmitters with special reference to nitric oxide: biological and chemical implications. *Reviews in Inorganic Chemistry* .2018;38(4) : 10.1515/revic-2018-0011 193-220 10.1515/revic-2018-0011

[2] : Nitric oxide boosters as defensive agents against COVID-19 infection: an opinion. *J Biomol Struct Dyn* .40(9) : 10.1080/07391102.2020.1852969 4285-4291 10.1080/07391102.2020.1852969 33251965PMC7754890

[3] : Nitric oxide as a therapeutic option for COVID-19 treatment: a concise perspective. *New Journal of Chemistry* .2021;45(4) : 10.1039/D0NJ03823G 1774-1784 10.1039/D0NJ03823G

# Peer review: Reviewer response for version 2

**DOI:** 10.5256/f1000research.78470.r97610

**Published:** 2021-10-25

**Authors:** Khosrow Kashfi

**Affiliations:** 1 Department of Molecular, Cellular and Biomedical Sciences, Sophie Davis School of Biomedical Education, City University of New York School of Medicine, New York, NY, 10031, USA

The authors have adequately addressed my original concerns. The revised submission is acceptable.

Is the review written in accessible language?

Yes

Are all factual statements correct and adequately supported by citations?

Yes

Are the conclusions drawn appropriate in the context of the current research literature?

Yes

Is the topic of the review discussed comprehensively in the context of the current literature?

Yes

Reviewer Expertise:

Pharmacologist with a strong background in biochemistry and intermediary metabolism.

I confirm that I have read this submission and believe that I have an appropriate level of expertise to confirm that it is of an acceptable scientific standard.

# Peer review: Reviewer response for version 1

**DOI:** 10.5256/f1000research.54420.r93464

**Published:** 2021-09-27

**Authors:** Khosrow Kashfi

**Affiliations:** 1 Department of Molecular, Cellular and Biomedical Sciences, Sophie Davis School of Biomedical Education, City University of New York School of Medicine, New York, NY, 10031, USA

Nitric oxide (NO) is one of the ten smallest molecules found in nature. It is released intracellularly when L-arginine is oxidized by the enzyme nitric oxide synthase (NOS), of which there are three isoforms. Neuronal (nNOS, NOS1) and endothelial (eNOS, NOS3) are constitutive calcium-dependent forms of the enzyme regulated by negative feedback and release low fluxes of NO over a short period regulating neural and vascular function, respectively. The third isoform (iNOS, NOS2) is calcium-independent, inducible, produces supra-physiological concentrations of NO, and is involved in immune surveillance. NO can also be produced through the reduction of nitrite/nitrate under low oxygen conditions.

 NO has important roles in normal physiology, exemplified by regulation of vascular relaxation, control of inflammation by inhibiting NF-κB activation, and suppression of pro-inflammatory mediators in mast cells, macrophages, and vascular smooth muscles. In addition, NO regulates blood flow, modulates platelet and leukocyte activation, adhesion, and aggregation.

 In this review, the authors have focused on NO's role in defense against multiple microbial pathogens [including viruses, bacteria, SARS-CoV (COVID-19), protozoa, and fungi/yeast]. In addition, they have highlighted the possibility that exogenous NO might have therapeutic potential as a broad-spectrum antimicrobial. Finally, they have also summarized various NO donating/releasing platforms.

 In general, I do not think that there is a "right" or "wrong" way of presenting a review, as long as it is accurate, balanced, and gives adequate historical background. I found this review to be quite compelling and of interest to those starting in this field and an update for the seasoned investigators. In short, it is an authoritative review. For the novice, the review gives the directions for further in-depth reading. Finally, I would like to make some suggestions to the text for the authors to consider as I believe it would add to the overall body of this work.

- Throughout the text, when you talk about “NO may be taken as dietary substrate (inorganic nitrate, L-arginine)….", please also discuss L-citrulline as a source of NO. We recently reviewed this topic and you may want to use this review as a source to expand on this ^1^.
- In Table 1, please add L-citrulline as a substrate and give the appropriate biochemical equation.
- Under Therapeutic inorganic nitrite and nitrate, please consider adding the use of nitrite/nitrate in animal models of wound healing. Refer to Afzali *et al*. 2020 ^2^ for an introduction to this.
- Under novel nitric oxide agents, please add RRx-001, which is a novel NO modulator ^3^.
- As summarized in the review, dietary intake of foods such as beetroot that are rich as a source of nitrate have many health benefits. And as mentioned, "Dietary nitrate has profound metabolic effects and appears to have the potential for reversing the metabolic syndrome and have anti-diabetic effects.". However, although the beneficial metabolic effects of inorganic nitrate and nitrite in type 2 diabetes mellitus have been documented in animal experiments, this is not the case for humans. Perhaps this should be mentioned on page 18 of the review ^4^.

Is the review written in accessible language?

Yes

Are all factual statements correct and adequately supported by citations?

Yes

Are the conclusions drawn appropriate in the context of the current research literature?

Yes

Is the topic of the review discussed comprehensively in the context of the current literature?

Yes

Reviewer Expertise:

Pharmacologist with a strong background in biochemistry and intermediary metabolism.

I confirm that I have read this submission and believe that I have an appropriate level of expertise to confirm that it is of an acceptable scientific standard, however I have significant reservations, as outlined above.

## References

[1] : Endogenous flux of nitric oxide: Citrulline is preferred to Arginine. *Acta Physiol (Oxf)* .231(3) : 10.1111/apha.13572 e13572 10.1111/apha.13572 33089645

[2] : Acidified nitrite improves wound healing in type 2 diabetic rats: Role of oxidative stress and inflammation. *Nitric Oxide* .2020;103: 10.1016/j.niox.2020.07.001 20-28 10.1016/j.niox.2020.07.001 32693171

[3] : From METS to malaria: RRx-001, a multi-faceted anticancer agent with activity in cerebral malaria. *Malar J* .2015;14: 10.1186/s12936-015-0720-5 218 10.1186/s12936-015-0720-5 26017006PMC4453052

[4] : Lost-in-Translation of Metabolic Effects of Inorganic Nitrate in Type 2 Diabetes: Is Ascorbic Acid the Answer?. *Int J Mol Sci* .2021;22(9) : 10.3390/ijms22094735 10.3390/ijms22094735 33947005PMC8124635

# Response

**Published:** 2021-10-14

**Authors:** Philip Bath

**Affiliations:** University of Nottingham, UK

**Reviewer 1 responses**

**We thank Dr. Kashfi for his most helpful and considered comments and respond to each as below.**

 In this review, the authors have focused on NO's role in defense against multiple microbial pathogens [including viruses, bacteria, SARS-CoV (COVID-19), protozoa, and fungi/yeast]. In addition, they have highlighted the possibility that exogenous NO might have therapeutic potential as a broad-spectrum antimicrobial. Finally, they have also summarized various NO donating/releasing platforms.

 In general, I do not think that there is a "right" or "wrong" way of presenting a review, as long as it is accurate, balanced, and gives adequate historical background. I found this review to be quite compelling and of interest to those starting in this field and an update for the seasoned investigators. In short, it is an authoritative review. For the novice, the review gives the directions for further in-depth reading. Finally, I would like to make some suggestions to the text for the authors to consider as I believe it would add to the overall body of this work.

- Throughout the text, when you talk about “NO may be taken as dietary substrate (inorganic nitrate, L-arginine)….", please also discuss L-citrulline as a source of NO. We recently reviewed this topic, and you may want to use this review as a source to expand on this ^1^ .

**We have added L-citrulline wherever dietary L-arginine is mentioned (abstract, text and tables), and added two references: Flam *et al. *2007, Bahadoran *et al.* 2021.**

- In Table 1, please add L-citrulline as a substrate and give the appropriate biochemical equation.

**We have added L-citrulline as a substrate and the relevant equations.**

- Under Therapeutic inorganic nitrite and nitrate, please consider adding the use of nitrite/nitrate in animal models of wound healing. Refer to Afzali *et al*. 2020 ^2^ for an introduction to this.

**We have added a comment relating to the reference of Afzali *et al.* 2020, and the reference itself.**

- Under novel nitric oxide agents, please add RRx-001, which is a novel NO modulator ^3^ .

**We have added a comment relating to RRx-001 using a reference from Oronsky *et al.* 2020, and the reference itself.**

- As summarized in the review, dietary intake of foods such as beetroot that are rich as a source of nitrate have many health benefits. And as mentioned, "Dietary nitrate has profound metabolic effects and appears to have the potential for reversing the metabolic syndrome and have anti-diabetic effects.". However, although the beneficial metabolic effects of inorganic nitrate and nitrite in type 2 diabetes mellitus have been documented in animal experiments, this is not the case for humans. Perhaps this should be mentioned on page 18 of the review ^4^ .

**We have added two references relating to beetroot juice improving insulin sensitivity and reducing blood glucose (Wootton *et al.* 2014, Beals *et al.* 2017).**
